# Supramolecular architectures and structural diversity in a series of lead (II) Chelates involving 5-Chloro/Bromo thiophene-2-carboxylate and N,N’-donor ligands

**DOI:** 10.1186/1752-153X-7-139

**Published:** 2013-08-15

**Authors:** Samson Jegan Jennifer, Packianathan Thomas Muthiah

**Affiliations:** 1 School of Chemistry, Tiruchirappalli, Tamil Nadu 620024, India

**Keywords:** Metal bite, Lone pair, Phenanthroline, 5-chlorothiophen-2-carboxylic acid, Pb(II), Halogen bonding

## Abstract

**Background:**

Lead is a heavy toxic metal element in biological systems and is one of the major pollutants as a result of its widespread use in industries. In spite of its negative roles the coordination chemistry of Pb(II) complexes is a matter of interest. The N,N’-bidentate aromatic bases such as BPY,4-BPY and PHEN (BPY = 2,2′bipyridine, 4-BPY = 4,4′-dimethyl-2,2′-bipyridine, PHEN = 1,10-Phenanthroline) are widely used to build supramolecular architectures because of their excellent coordinating ability and large conjugated system that can easily form π-π interactions among their aromatic moieties. A series of novel Pb(II) complexes in concert with 5-CTPC, 5-BTPC (5-CTPC = 5-chlorothiophen-2-carboxylate, 5-BTPC = 5-bromothiophen-2-carboxylate) and corresponding bidentate chelating N.N′ ligands have been synthesized and characterized.

**Results:**

Five new Pb (II) complexes [Pb(BPY)(5-CTPC)_2_] (1), [Pb(4-BPY)(5-CTPC)_2_] (2), [Pb_2_(PHEN)_2_(5-CTPC)_4_] (3), [Pb(4-BPY)(5-BTPC)_2_] (4) and [Pb_2_(PHEN)_2_(5-BTPC)_2_(ACE)_2_] (5) have been synthesized. Even though in all these complexes the molar ratio of Pb, carboxylate, N,N-chelating ligand are the same (1:2:1), there is a significant structural diversity. These complexes have been characterised and investigated by elemental analysis, IR, ^1^H-NMR,^13^C-NMR, TGA, and photoluminescence studies. Single crystal X-ray diffraction studies reveal that complexes (1, 2) and (4) are mononuclear while (3 and 5) are dinuclear in nature which may result from the chelating nature of the ligands, various coordination modes of the carboxylates, and the coordination geometry of the Pb(II) ions.

**Conclusions:**

The observation of structures 2,4 and 3,5 show the structural changes made just chloro/bromo substituent of the thiophene ring. A detailed packing analysis has been undertaken to delineate the role of valuable non covalent interactions like X…π, H…X, (X = Cl/Br). A quadruple hydrogen bond linking the monomeric units and generating a supramolecular architecture is observed in (1). The metal bite unit comprised of PbN_2_C_2_ (*i.e.* Pb-N-C-C-N-Pb) is the repeating unit in all the five complexes and they have almost same geometrical parameters. This metal bite has been identified as the self assembly unit in complexes.

## Background

The molecular self assembly with discrete supramolecular units directed by metal ligand coordination and weak interactions are of considerable interest [1-6]. The interplay of coordination motifs and supramolecular synthons are of current interest to generate novel materials (metal organic frameworks (MOF’s), coordination polymers, inorganic hybrid materials, etc.). When a sensible choice of the central metal ion and a ligand whose favourite coordination modes are known one can design an anticipated variety of supramolecular motifs [6-10].

**Scheme 1:**
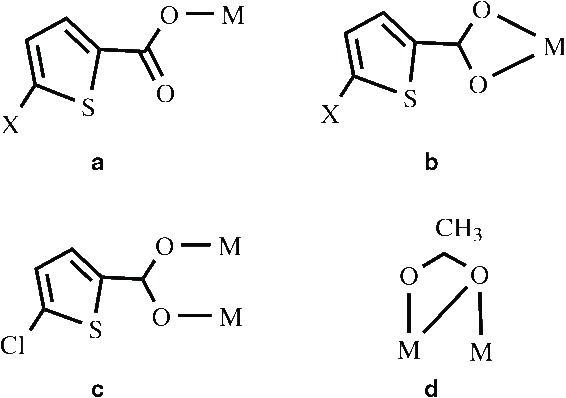
**Coordination modes of 5-CTPC, 5-BTPC and acetate. (a)** X = Cl in 4 **(b)** X = Cl, Br in 2 and 4 respectively, where M = Pb.

Lead is a heavy toxic metal element in biological systems and is one of the major pollutants as a result of its widespread use in industries. In spite of its negative roles the coordination chemistry of Pb(II) complexes is a matter of interest, which display interesting structural features as a consequence of the large radius, adoption of different coordination numbers from 2 to 10 and especially based on the extent to which the lone pair is stereo chemically active [11-15]. A common strategy followed in designing of the Pb(II) complexes is the presence of carboxylate and bipyridne systems in the coordination sphere [16-33]. Lead(II) carboxylate systems containing mono, di and polycarboxylate have been extensively used in the construction of metal organic networks [23-33]. The N,N’-bidentate aromatic bases such as BPY,4-BPY and PHEN are widely used to build supramolecular architectures because of their excellent coordinating ability and large conjugated system that can easily form π-π interactions among their aromatic moieties, which are either intra or intermolecular [16-22]. The thiophene carboxylates possess various coordination modes such as monodentate, bidentate chelating, bidentate bridging when coordinated to the metal ion (Scheme 1). The rigid heterocyclic five member thiophene ring possesses distinct physical and chemical properties [34,35]. Also in this heterocyclic thiophene ring the relatively bigger size of the sulphur atom makes the possibility for delocalisation of its lone pair of electrons, which results in good charge transfer ability of the ligand [35].

Our strategy is to investigate the chelating behaviour of these ligands towards lead (II) along 5-CTPC and 5-BTPC. In all the five complexes BPY, 4-BPY and PHEN act as a nitrogen donor ligand forming 3D supramolecular architectures in the solid state. Although there are some reports of diverse coordination polymers involving multicarboxylate ligands containing S atom and bidentate chelating ligands like PHEN, 2-BPY as well as bridging ligand such as 4-4′-bipyridine as coligands [36-41], there are only a few reports involving the halo substituted thiophene carboxylate ligand for the construction of organic–inorganic hybrids [42]. 5-CTPC not only shows versatile coordination modes but also exhibits non covalent interactions like Cl…π and C-H…Cl [42-45]. In our present investigation of five Pb(II) complexes, we have observed the different coordination number, and different coordination modes of the carboxylate ligand which gives the idea of constructing different supramolecular architectures. The coordination modes of the carboxylate group, observed in these Pb(II) complexes are shown in scheme.1. In addition a previously reported complex [Pb(TPC)_2_(PHEN)] (TPC = Thiophene 2- carboxylic acid) has been compared with structures (3, 5) [46].

## Results and discussion

### Geometry around lead

**Figure 1:**
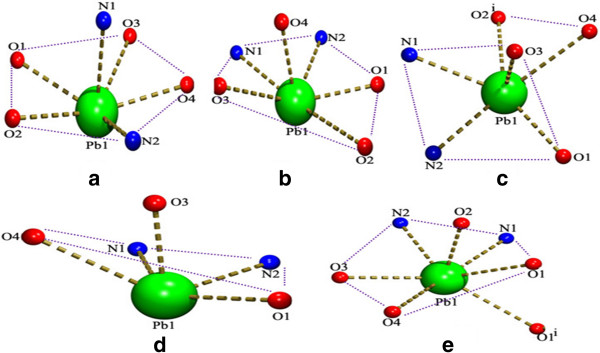
**Comparison of coordination environment around the Pb(II) ions showing the geometries. (a)** Complex 1; **(b)** Complex 2; **(c)** Complex 3; **(d)** Complex 4 and **(e)** Complex 5.

The lone pair of electrons has a great influence on the structure of the complex [11,47,48]. In the coordination chemistry of the Pb(II) ion, the terms holo and hemi directed are used to describe the geometries around the central Pb atom. Pb(II) complexes where the bonds to ligand atoms are placed throughout the surface of the encompassing globe are said to be holo directed, while hemidirected refers to those cases in which the bonds to ligand atoms are directed throughout only part of an encompassing globe [49]. The coordination numbers of lead in complexes 1 and 2 are six, and they exhibit a pentagonal pyramidal geometry. While the coordination numbers of 4 and 5 are five and seven respectively, and they show distorted square pyramidal and distorted pentagonal bipyramidal geometry respectively. Comparison of coordination modes of the carboxylates are listed in (Table 1). The structure 3 shows an unusual capped square pyramidal geometry. In complex 1, the axial position of the pentagonal pyramid is occupied by the N1 of the BPY by which it differs from 2 where this position is occupied by O4 of the 5-CTPC (Figure 1). In 4 the axial position is occupied by O3 of the 5-BTPC. The coordination geometry of complexes 1-3,4 as well as the Pb-O and the Pb-N bond directions show a gap around the Pb(II) ion, occupied possibly by a stereoactive lone pair of electrons on lead(II). The coordination around the lead atoms is hemidirected with a significant gap trans to chelating BPY, 4-BPY and PHEN ligands (Figure 1). In 5 PHEN is a space demanding ligand upon coordination with the Pb(II) ion it forms a seven coordinated holodirected distorted pentagonal bipyramidal complex instead a hemidirected one with less coordination number. The hemidirected geometry is the most preferred for intermediate coordination numbers between 6-8 [50]. There is a π–π stacking interaction, the parallel aromatic rings belonging to adjacent chains in complexes 1, 2 and 3 that may help to increase the ‘gap’ in the coordination geometry around the Pb(II) ion.

**Table 1:** Comparison of coordination modes of various carboxylates

**Complex**	**Caboxylate**	**Type of coordination**	**Geometry**
1	5-CTPC	Bidentate chelating	Pentagonal pyramidal
2	5-CTPC	Bidentate chelating	Pentagonal pyramidal
3	5-CTPC	Bidentate chelating, Bidentate bridging	Capped square pyramidal
4	5-BTPC	Bidentate chelating, Monodentate	Distorted square pyramidal
5	5-BTPC	Bidentate chelating	Pentagonal bipyramidal
	ACE	Bidentate chelating bridging	

### Crystal structure description of [Pb(BPY)(5-CTPC)_2_] (1)

**Figure 2:**
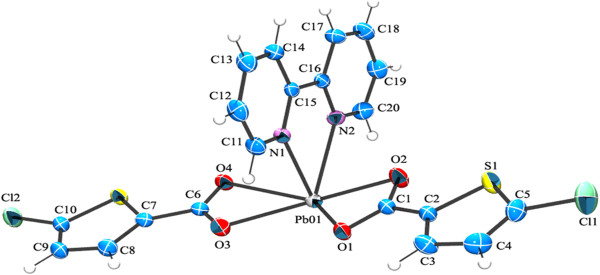
ORTEP of complex 1 showing the atom-numbering scheme with displacement ellipsoids drawn at 50% probability level for all non hydrogen atoms and H atoms are shown as small spheres of arbitrary radii.

Single-crystal X-ray diffraction analysis reveals that complex 1 consists of a monomeric Pb(II) ion, a BPY and two bidentate (5-CTPC) molecules as shown in (Figure 2). Each Pb(II) ion is a hexa coordinated environment with two nitrogen from a chelating BPY molecule with a Pb-N bond distances (2.481(2) Å and 2.595(2)Å) and four oxygen atoms from two bidentate 5-CTPC molecules with the Pb-O bond lengths ranging from2.532(2) Å to 2.624(2) Å.

**Figure 3:**
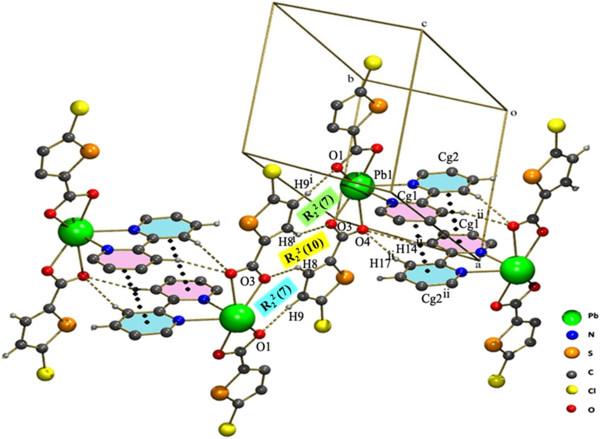
**View of 1 showing the 1D chain made up of C-H…O interactions and the π –π stacking inetractions.** The hydrogen atoms which are not involved in hydrogen bonding are omitted for clarity.

**Figure 4:**
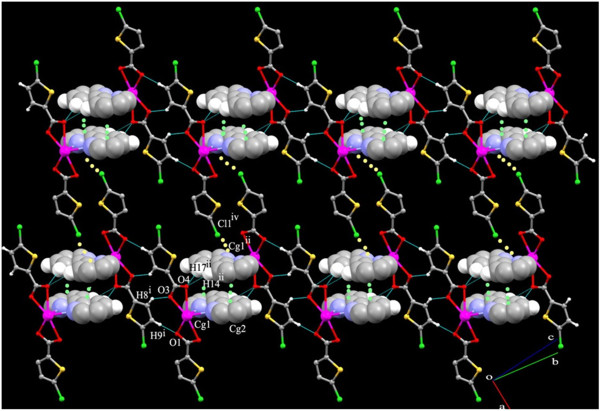
Crystal packing in 1 showing the Cl…Cg interactions.

The monomeric units are strongly linked to each other by a quadruple hydrogen bonding motif consisting of four C-H. . .O hydrogen bonding interactions (inbetween the hydrogens of the thiophene ring and oxygen from the bidentate-chelating coordinated carboxyl) (Table 2, Figure 3). The quadruple hydrogen bonds fused by three rings, which can be represented by the graph-set notation R_2_^2^(7),R_2_^2^(10),R_2_^2^(7) (Figure 4) [51]. Thus it forms a dimer made of quadruple hydrogen bonds. Surprisingly inbetween the adjoining units there is a weak coordination bond (PbO1-O1 = 3.060 Å). A similar structural unit with same Pb-O weak coordination bond has been reported earlier [46].

**Table 2:** Hydrogen bond metrics for complexes 1-5

**D---H....A**	**H…A (Ǻ)**	**D…A (Ǻ)**	∟**D -H…A**	**Symmetry operation**
[Pb(BPY)(5-CTPC)2] (1)				
C8-H8…O3	2.5300	3.334(4)	145.00	3-x,1-y,2-z
C9-H9…O1	2.4200	3.318(4)	163.00	3-x,1-y,2-z
C14-H14…O4	2.4700	3.364(4)	162.00	2-x,-y,1-z
C17-H17…O4	2.4700	3.351(4)	158.00	2-x,-y,1-z
C18-H18…O2	2.5300	3.273(4)	137.00	1-x,-y,1-z
[Pb(4-BPY)(5-CTPC)2] (2)				
C9-H9…O2	2.56	3.437(4)	157	2-x,1-y,-z
[Pb2(PHEN)2(5-CTPC)4] (3)				
C4-H4…Cl1	2.8100	3.617(4)	146.00	−2-x,-y,-z
C11-H11…O2	2.5000	3.143(5)	127.00	-x,1-y,1-z
[Pb(4-BPY)(5-BTPC)2] (4)				
C22-H22A…Br1	2.8700	3.791(5)	162.00	2-x,-y,2-z
C9-H9…O2	2.5600	3.424(6)	155.00	2-x,1-y,1-z
[Pb2(PHEN)2(5-BTPC)2(ACE)2] (5)				
C8-H8…O1	2.6000	3.325(5)	135.00	2-x,1-y,1-z
C8-H8…O4	2.4600	3.149(8)	130.00	2-x,1-y,1-z
C15-H15…O2	2.5200	3.109(7)	121.00	2-x,1-y,2-z

Thus these dimers are further interdigited to each other by the π- π stacking interactions between the bipyridine rings leading to one dimensional infinite chains Cg1 → Cg2^ii^ and Cg2 → Cg1^ii^ [Symmetry code ii: 2-X,-Y,1-Z] (Cg1 = N1,C11-C15 and Cg2 = N2,C16-C20) (Figure 3). Further the chains are connected to each other by the Cl∙∙∙π interaction found inbetween the Cl1 of the five member thiophene ring involving (S1,C2-C5) and the six membered N-hetero ring of the BIPY involving (N1,C11-C15) (Figure 4).

### Crystal structure description of [Pb(4-BPY)(5-CTPC)_2_] (2)

**Figure 5:**
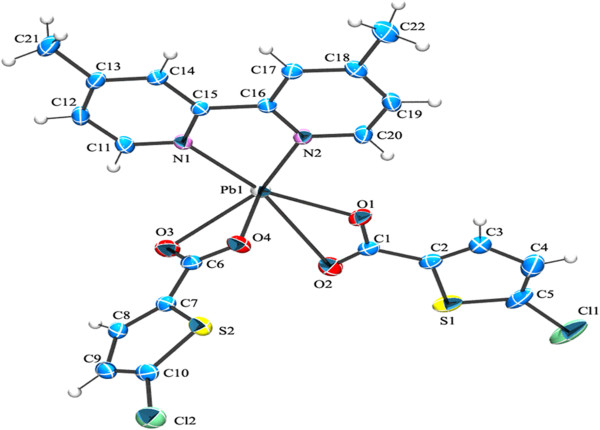
**ORTEP of complex 2 showing the atom-numbering scheme.** Displacement ellipsoids are drawn at the 50% probability level for all non hydrogen atoms and H atoms are shown as small spheres of arbitrary radii.

The core structure of **2** is very similar to that of 1 and it consists of one Pb(II) ion, a 4-BPY and two bidentate (5-CTPC) molecules as shown in (Figure 5). Each Pb(II) ion has a hexa coordinated environment with two nitrogen from a chelating 4-BPY molecule with a Pb-N bond distances (2.593(2) Å and 2.626(2) Å) and four oxygen atoms from two bidentate 5-CTPC molecules with the Pb-O bond lengths ranging from (2.3469(19) Å to 3.019(2) Å). Although the coordination geometry of lead (II) ion is same for both complexes 1 and 2, they differ in the atoms coordinated at the apical site of the pentagonal pyramid. In 1 it is occupied by the N1 (Pb-N1 2.481(2) Å) of the bidentate BPY ligand while in 2 it is occupied by the O4 (Pb-O4 2.3469(19) Å) of the 5-CTPC.

**Figure 6:**
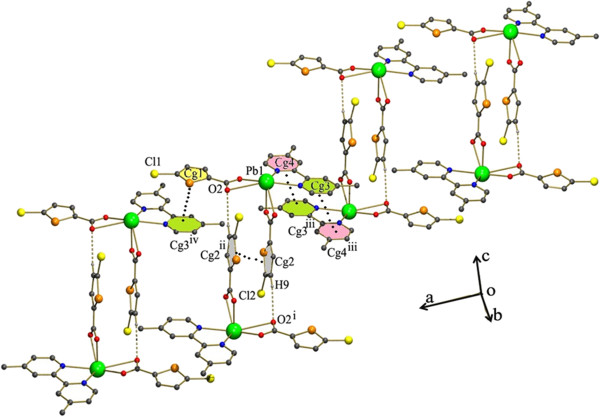
Perspective view of 2 showing the I shaped dimer formed by C-H…O and π –π stacking interactions.

The monomeric units are linked by C-H. . .O interaction between C9-H9…O2^i^ [Symmetry code i: 2-x,1-y,-z] and π –π stacking interactions inbetween two five member rings Cg2 → Cg2^ii^ where Cg2 = S2, C7-C10 [symmetry code ii: 2-X,1-Y,-Z] (Figure 6). Thus these interactions forms I shaped dimers (Figure 6). This I shaped dimer is nailed to the two other I shaped dimer on its adjacent sides by two sets of π –π stacking interactions (Cg1 → Cg3^iv^ on one side and Cg3 → Cg4^iii^, Cg4 → Cg3^iii^ on the other side). Another pi-pi stacking is found between the two six member rings of the two adjacent 4-BPY ligands.

### Crystal structure of description of [Pb_2_(PHEN)_2_(5-CTPC)_4_] (3)

**Figure 7:**
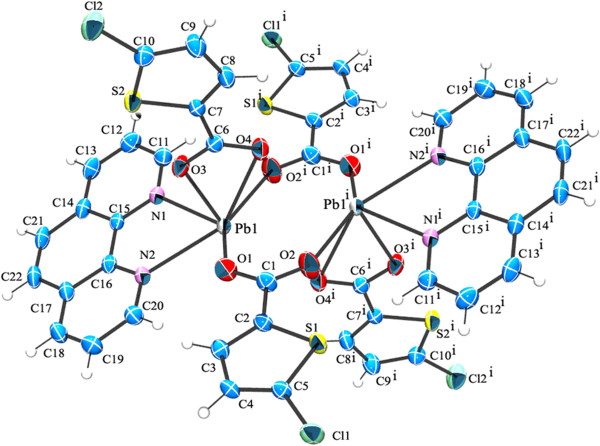
**ORTEP of complex 3 showing the atom-numbering scheme.** Displacement ellipsoids are drawn at the 50% probability level for all non hydrogen atoms and H atoms are shown as small spheres of arbitrary radii.

The complex 3 contains a dinuclear lead unit with two carboxylate groups bridging the lead atoms (Pb-O-C-O-Pb link). The coordination sphere of each Pb is completed by the bidentate chelating ligands, PHEN and 5-CTPC. The coordination around each Pb can be best described as capped square pyramidal geometry (Figure 1c, Figure 7).

**Figure 8:**
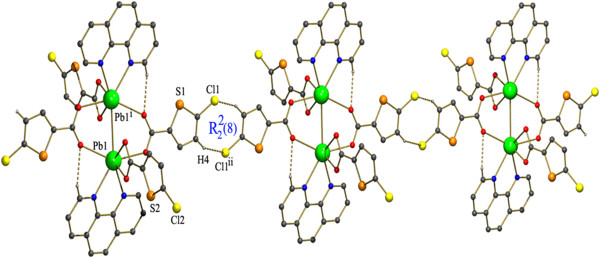
**Hydrogen bond pattern in 3 showing the formation of 1D chain by C-H.** Cl interactions and formation of the R_2_^2^(8) graphset motifs inbetween the individual dimeric units.

The Pb-N bond distances are 2.604(2)Å to 2.614(3) Å, whereas the Pb-O bond distances ranges from 2.332(2)Å to 2.813(2)Å. Of the four oxygen atoms two are from a bidentate carboxylate of 5-CTPC and other two are bridging oxygens from two different 5-CTPC. The arrangement of the PHEN and carboxylate ligands suggests a gap coordination around the Pb(II) ion, which might be occupied by a stereoactive lone pair of electrons on Pb(II) ion and also probably due to steric hindrance of the PHEN ligand. Columns of lead atoms with Pb---Pb separation of 3.7020(2) bridged by two the 5-CTPC carboxylates extend along the crystallographic a axis. The individual dimeric unit of the one layer are almost parallel to each other of the next layer and further the dimeric units are linked to each other by C28-H28. . .Cl1^ii^ (Symmetry code (ii) :-2-x,-y,-z) interaction between the thiophene ring of two dimers. This interactions thus form an 8 member ring (Figure 8) which can be represented by the graph-set motif R_2_^2^(8) [51]. This forms a chain extending along the crystallographic a axis.

**Figure 9:**
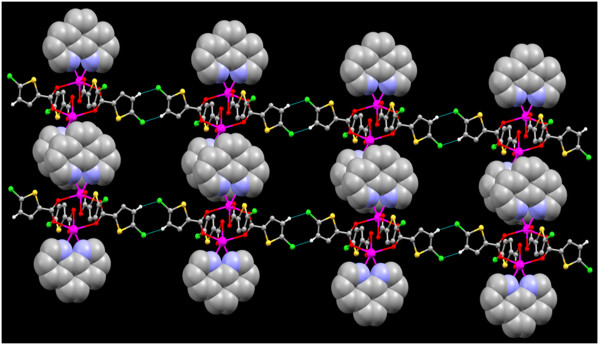
View of complex 3 along the crystallographic c axis showing the π- π stacking interactions (spacefilled model) inbetween the 1D chains.

Two of these chains are interlocked to each other by the π- π stacking interactions between the two 6 membered rings made up of the nearby 1,10 phenanthroline rings Cg5 → Cg5^iv^ (Cg5 = C14, C15,C16,C17,C22 & C21) [Symmetry code (iv) :-X,2-Y,1-Z]. The Cl of the two thiophene rings plays a vital role in stabilisation of crystal structure, three sets of Cl…Cg interactions are found inbetween C5-Cl1…Cg3^i^, C5-Cl1…Cg5^ii^, C10-Cl2…Cg1^iii^; where Cg1 = S1,C2-C5, Cg3 = N1,C11-C15 and Cg5 = C14-C17,C22,C21 [symmetry code i = -1 + X,-1 + Y,-1 + Z; ii = -1-X,1-Y,1-Z; iii = X,Y,1 + Z] (Figure 9). Similarly a C-H…π interaction is found inbetween the H of the PHEN ligand and the five membered thiophene ring. C19-H19…Cg2 [Cg2 = S2,C7-C10 symmetry code: -1-X,1-Y,1-Z].

This complex 3 can be structurally compared with a previously reported in the literature [Pb(TPC)_2_(PHEN)] (TPC = Thiophene2-carboxylic acid) [46]. It is interesting to note that the reported Pb-TPC complex has a monomeric and these units are linked to one another by stacking and weak coordination bonds whereas complex 3 has a dinuclear unit. The only difference between the two complexes is the chloro substitution present in complex 3.

### Crystal structure description of [Pb(4-BPY)(5-BTPC)_2_] (4)

**Figure 10:**
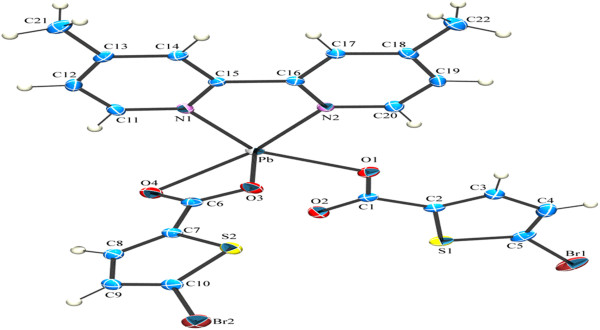
**ORTEP of complex 4 showing the atom-numbering scheme.** Displacement ellipsoids are drawn at the 50% probability level for all non hydrogen atoms and H atoms are shown as small spheres of arbitrary radii.

Complexes 2 and 4 differ only by the chloro/bromo substitution in the thiophene ring. Hence they are near isomorphous with similar cell parameters (Table 3). Regarding the coordination geometry, in 4 one carboxylate is bidentate chelating while the other is in monodentate fashion. Whereas in 2 both are in bidentate fashion. Thus in 4 the Pb(II) ion is pentacoordinated, with two N atoms of the (4-BPY), one O atom of the bidentate chelating carboxylate and one O of the monodentate carboxylate in the basal plane and one O from the bidentate chelating carboxylate completing the distorted square-pyramidal geometry (Figure 10).

**Table 3:** Crystal data and refinement parameters for complexes 1-5

	**Complex 1**	**Complex 2**	**Complex 3**	**Complex4**	**Complex5**
Empirical formula	C20H12N2O4Cl2S2Pb	C22H16Cl2N2O4S2Pb	C44H24Cl4N4O8S4Pb2	C22H16Br2N2O4PbS2	C38H26Br2N4O8Pb2S2
Formula weight	686.56	714.61	1421.15	803.51	1304.97
Temp, K	296	296	296	296	296
λ (Å)	0.71073	0.71073	0.71073	0.71073	0.71073
Crystal system	Triclinic	Triclinic	Triclinic	Triclinic	Monoclinic
Space group	P-1	P-1	P-1	P-1	P21/c
a (Å)	9.0760(1)	10.6261(3)	10.2998(1)	10.710(5)	8.918(5)
b (Å)	12.0102(2)	10.9835(2)	11.3830(1)	10.973(5)	23.841(5)
c (Å)	12.0754(2)	11.4710(2)	11.4287(1)	11.623(5)	12.744(5)
α(º)	116.441(1)	115.441(1)	100.292(1)	115.592(5)	90
β (º)	93.682(1)	98.773(1)	103.011(1)	98.676(5)	133.39(2)
γ (º)	106.176(1)	96.052(1)	115.391(1)	96.238(5)	90
V (Å3)	1104.46(3)	1172.71(5)	1120.29(2)	1194.5(9)	1969.0(16)
Z	2	2	1	2	2
ρ calcd (g/cm3)	2.065	2.024	2.106	2.234	2.201
μ (mm-1)	8.099	7.632	7.989	10.615	10.732
F(000)	652	684	676	756	1224
Crystal size (mm)	0.04 × 0.05 × 0.05	0.05 x 0.05 × 0.06	0.04 × 0.05 × 0.06	0.08 × 0.09 × 0.06	0.09 × 0.05 × 0.06
No of reflections collected	7170	8554	7529	8114	6592
Number restraints	0	0	0	0	0
Goodness-of-fit on F2	1.026	1.03	1.00	1.02	0.98
Final R1 index [I > 2σ(I)]	0.0239	0.0240	0.0303	0.0290	0.0309
wR2 (all data)	0.0541	0.0556	0.0635	0.0633	0.0690
Largest difference in peak and hole (e Å-3)	−0.61, 1.14	−0.63, 1.27	−0.45, 1.18	−1.08, 1.13	−1.05, 0.88
CCDC number	821366	821367	821365	920520	920521

R1 = ∑(||Fo|-|Fc||)/∑| Fo |; wR2 = [∑w(|Fo|-|Fc|2)2]/∑w(|Fo|2)]1/2.

**Figure 11:**
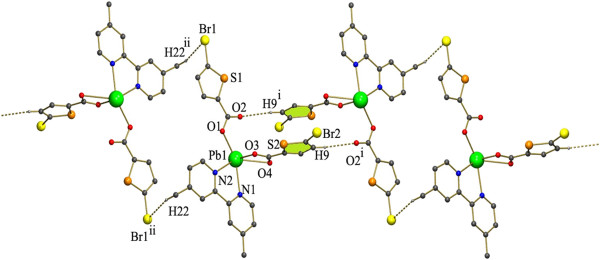
1D H-bonded chain formed by C-H…Br, C-H…O and π- π stacking interactions existing in 4.

Although the first order coordination of 4 is different from that of 2, the same kind of C-H…O and π- π stacking interactions are found in 4. The monomeric units are linked to each other by the π- π stacking interactions in between the two five membered thiophene rings Cg1 → Cg1^i^ (Cg1 = S2,C7-C10) [symmetry code i: 2-X,1-Y,1-Z]. The C-H…O interactions found inbetween C9-H9…O2^i^ [symmetry code i: 2-X,1-Y,1-Z]. This leads to the formation of I shaped dimer as in 2. This dimeric unit is further linked to each other to form the tetrameric unit by a pair of C-H…Br interactions (Figure 11) while these kind of interactions were absent in 2.

**Figure 12:**
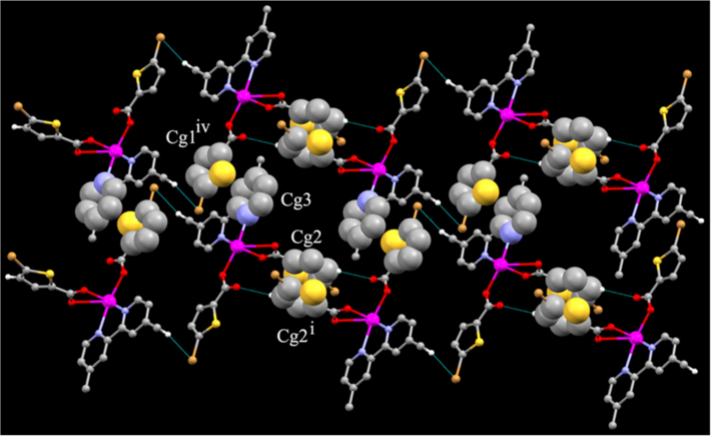
Crystal packing in 4 the rings involved in stacking interactions are shown as spacefilled models.

Thus they form a chain. Each of these chains are further linked by the the π- π stacking interactions in between the five membered thiophene rings and a N containing heterocyclic ring of the 4-BPY Cg3 → Cg1^iv^ (Cg3 = N1, C11- C15 and Cg1 = S1,C2-C5) (Figure 12). The most unusual feature of 4 is the monodentate coordination of the carboxylate group leading to a variety of changes in the 3Dstructure. The reason for the monodentate coordination of the 5-BTPC may be attributed to the strong C-H…Br and C-H…O interactions involving the 5-BTPC atoms (Figure 11).

### Crystal structure of description of [Pb_2_(PHEN)_2_(5-BTPC)_2_(ACE)_2_] (5)

**Figure 13:**
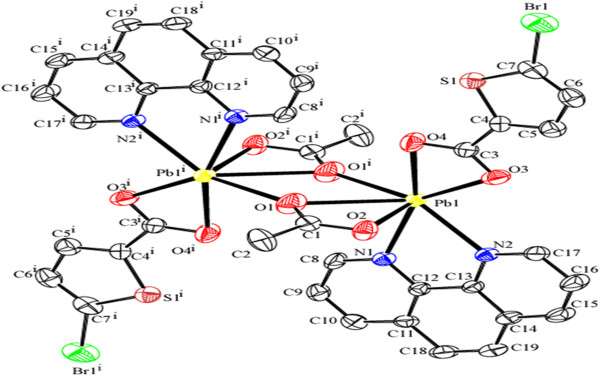
**ORTEP of complex 5 showing the atom-numbering scheme.** Displacement ellipsoids are drawn at the 50% probability level for all non hydrogen atoms and H atoms are shown as small spheres of arbitrary radii.

As anticipated the usage of PHEN ligand produced a dinuclear structure as 3 (Figure 13). The central Pb(II) ion not only differs in primary coordination from 3 but it is entirely different from all the above structures. Each of the Pb(II) ion is coordinated to a PHEN and 5-BTPC in a bidentate chelating mode. Unlike the complex 3 the bridging carboxylate is replaced by chelating bridging acetate group of the starting material. This is also expected as the increase in Pb…Pb separation of 4.482(3) Å compared with that of 3.

**Figure 14:**
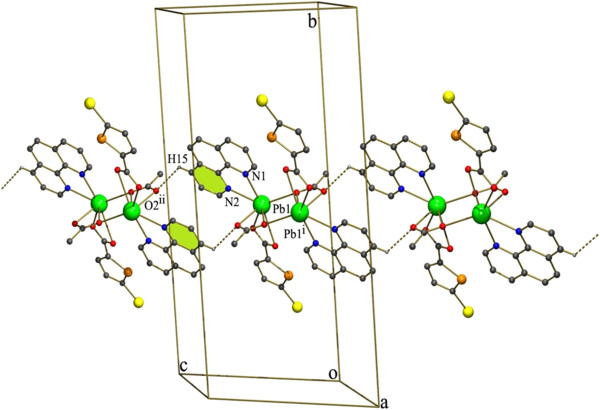
H-bonded chain formed by C-H…O and π- π stacking interactions existing in complex 5.

The expected C-H…Br and Br…Cg interactions are absent since one of the carboxylate has been replaced by the acetate group (Figure 14). The less steric hindrance of the acetate group made it possible for the C-H…O interaction inbetween H of the PHEN ring and O of the acetate group (C15-H15…O2^ii^ [symmetry code ii: 2-x,1-y,2-z]). Also as expected the chains formed by the C-H…Cl interactions (Figure 8) in the case of 3 is absent here. Further the individual dinuclear units are linked by the π- π stacking interactions are found in between the two 6 membered rings of the nearby PHEN rings Cg5 → Cg5^ii^ (Cg5 = N2, C13-C17) [Symmetry code (ii): 2-x,1-y,2-z]. These C-H…O and π- π stacking interactions extend along the crystallographic c axis leading to formation of a chain.

**Figure 15:**
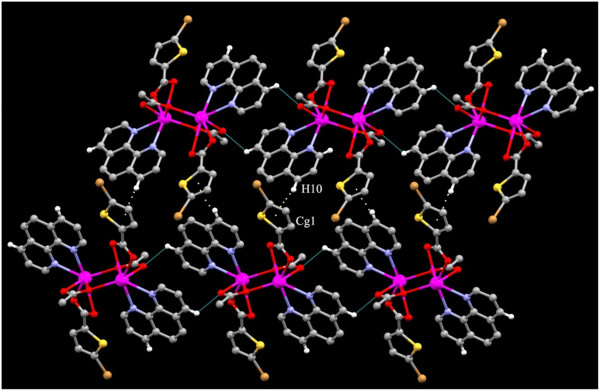
Perspective view of 5 the C-H…π interactions shown as dotted lines.

Two of these adjacent chains are interlinked by C-H…Cg interactions inbetween the H of PHEN ring of a chain and five membered thiophene ring of the adjacent chain [C10-H10…Cg1^ii^ symmetry code ii: 2-X,1/2 + Y,3/2-Z] (Figure 15).

### Involvement of X (X = Cl, Br) in interactions

**Figure 16:**
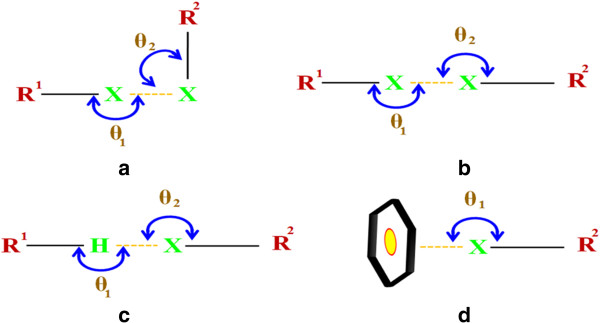
Various possible noncovalent interactions involving the halide, in (a) θ1 = θ2 ≠ 90; (b) θ1 = θ2 = 90 and (c,d) other possible interations.

Amid other non covalent interactions involving hetero atoms the role of Cl∙∙∙π, C-H ∙∙∙π, & Br∙∙∙π have been given importance due to their role in lattice stabilization and their contribution in determining the modes of packing in a molecular packing and in solid state it can never be over looked [52]. The non covalent interactions involving halogen fall in four types as given in (Figure 16a-d). The geometry of this R-H…X interaction play a major role in predictability of these interactions. In this backdrop it is worth in mentioning that we have studied various non covalent interactions involving the halogen atom in complexes 1-5 and the results are given in (Table 4). Various θ1 values have been observed with the highest C-Cl∙∙∙π for complex 1. Previous reports state that an interaction of the type H…X-C the preferred angle would be in the range of 90-130° rather than higher angles [53,54]. The observed θ2 values agree well with the above survey.

**Table 4:** Bond metrics for X (X = Cl,Br) involved non covalent interactions in complexes 1-5

**Complex**	**Presence of X (X = Cl,Br) involved non covalent interactions**	**Type of interaction**		**Distance in Å**	**θ1(°)**	**θ 2(°)**
1	√	(d) C-Cl∙∙∙π	C5 -Cl1∙∙∙ Cg3	3.5167 (19)	173.61 (15)	
2,5	Not involved					
3	√	(c) C-H∙∙∙Cl	C4-H4∙∙∙Cl1	2.81	146.42	130.75
		(d) C-Cl∙∙∙π	C5-Cl1∙∙∙Cg3	3.8566 (18)	93.99 (12)	
		(d) C-Cl∙∙∙π	C5-Cl1∙∙∙Cg5	3.9010 (18)	65.46 (12)	
		(d) C-Cl∙∙∙π	C10-Cl2∙∙∙Cg1	3.8632 (19)	131.18 (15)	
4	√	(c) C-H∙∙∙Br	C22-H22A∙∙∙Br1	2.8700	162.19	71.83
		(d) C-Br∙∙∙π	C(5)-Br(1)∙∙∙Cg(4)	3.462 (2)	86.36(13)	

### Repeating metal bite unit comprised of PbC_2_N_2_ in (1-5)

The metal bite (PbN_2_C_2_) unit is the repeating common unit of 1-5. This metal bite in 1 is formed with two coordinating N atoms, two carbon atoms of the BPY and the central lead atom, while in 2,4 it is formed by two coordinating N atoms, two carbon atoms of the 4-BPY and the central lead atom. While in 3,5 it is formed with two coordinating N atoms, two carbon atoms of the PHEN and the central lead atom.

**Figure 17:**
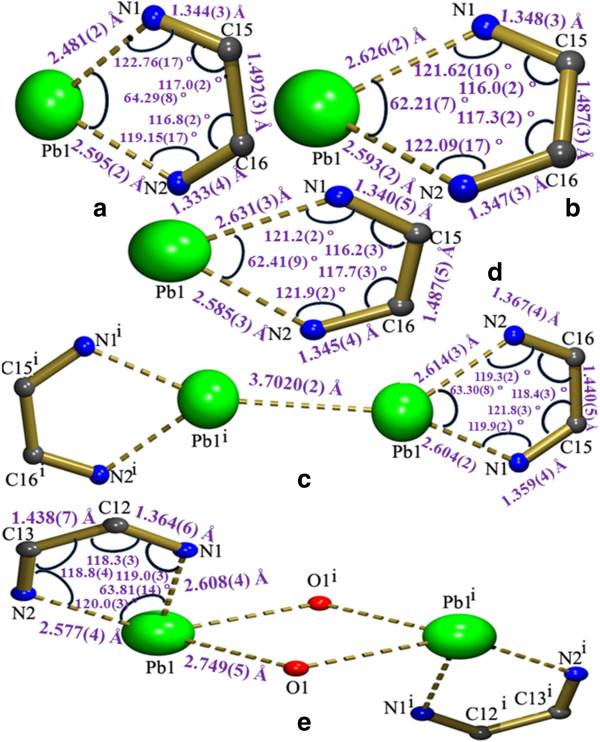
**Showing the metal bite which is the repeating. (a)** Complex 1; **(b)** Complex 2; **(c)** Complex 3; **(d)** Complex 4 and **(e)** Complex 5.

**Figure 18:**
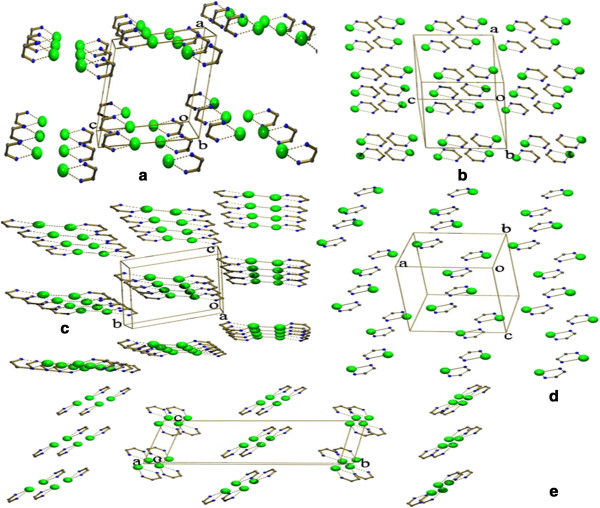
**The self assembly of the metal bites generating supramolecular architecture. (a)** Complex 1; **(b)** Complex 2; **(c)** Complex 3; **(d)** Complex 4 and **(e)** Complex 5.

The metal bite is a monomeric unit in 1, 2 and 4 while it is dimeric in 3,5. In 3 the metal bites are separated by a distance of 3.702(2) Å. While in 5 the metal bites are bridged by one of the carboxylate oxygen atoms. Interestingly the bond angles and bond lengths of the metal bite 1, 2 and 4 as well as 3 and 5 are almost comparable in complexes (Figure 17a,b,d) and (Figure 17c,e) respectively. On seeing the packing arrangement of these metal bite units, they are self assembled irrespective of whether they are monomeric in 1, 2 and 4 or dimeric in 3, 5. A view of the metal bites is shown in (Figure 18a-e). The dihedral angles between the two pyridine rings in 1, 2 and 4 are 4.69(15), 1.23(12) ° and 1.43(15) ° respectively. This shows that the planarity of BPY, 4-BPY and PHEN is maintained.

### Thermal analysis

**Figure 19:**
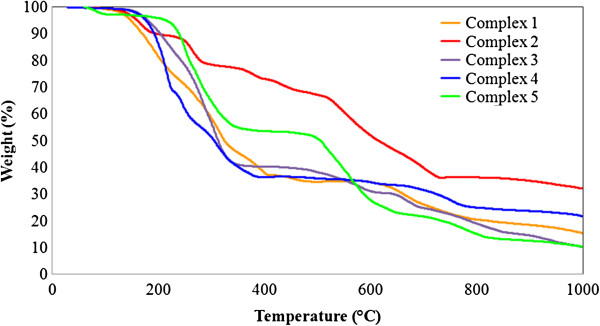
Thermograms of complexes 1-5 showing TGA curves at the heating rate of 10°C/min.

Thermogravimetric analysis (TGA) experiments of the complexes 1-5 were conducted under a static atmosphere of nitrogen at temperatures ranging from RT (room temperature) to 1000°C in order to determine the thermal stabilities (Additional file 1). Due to similarities of 1, 2, 4 and 3, 5 they show similar decomposition patterns (Figure 19). The complexes 1-5 started to melt well above 150°C showing a very small thermal effect. Complexes 1, 2, 4 showed two steps of thermal decomposition at (148-430°C, 150-540°C, 168-662°C) which probably due to two 5-TPC ligands. The decomposition of the bipyridine ligands took place at (435-957°C, 545-745°C, 667-842°C) in 1, 2 and 4 respectively. Complexes 3, 5 exhibit their first weight loss at temperature ranges of (145-405°C and 212-497°C). This is due to loss of four 5-TPC molecules in 3 as well as two 5-TPC molecules and two ACE molecules in 5. The second stage of decomposition at (416-848°C, 501-733°C) in 3 and 5 respectively are due to the decomposition of PHEN ligands.

### Photoluminiscent properties

**Figure 20:**
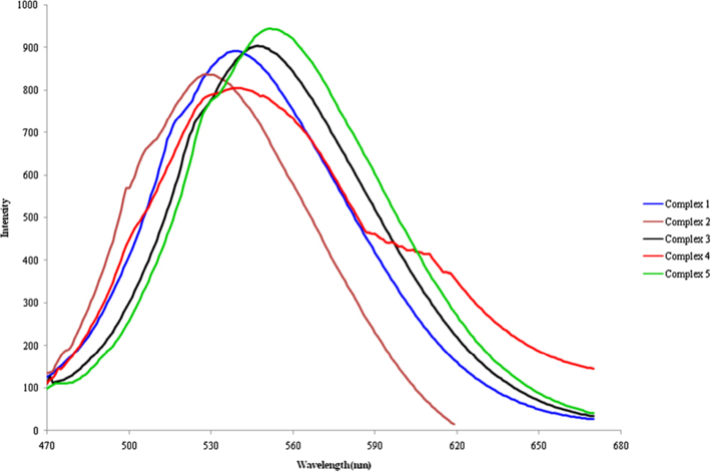
Solid-state emission spectra of complexes 1-5.

Previous studies show that Pb(II) complexes constructed with conjugated organic ligands can act as potential candidates for photoactive materials with appealing photoluminescence properties. Therefore, solid-state photo luminescence properties of Pb(II) complexes 1-5, with that of N,N’ chelating ligand, were investigated at room temperature based on their UV–vis spectra (Figure 20). The complex 1 displays a fluorescent emission at 538 nm (λ_ex_ = 423 nm), 2 at 527 nm (λ_ex_ = 436 nm), 3 at 547 nm (λ_ex_ = 441 nm), 4 at 540 nm (λ_ex_ = 429 nm) and 5 at 551 nm (λ_ex_ = 443 nm). The intense emission bands of complexes 1-5 to their corresponding ligands implies that the emission peaks of 1**-**5 may be due to a metal-to-ligand charge transfer (MLCT) and/or ligand-to-metal charge transfer (LMCT). Hence complexes 1-5 may serve as potential organic–inorganic hybrid photoactive materials [55].

## Experimental

### Materials and methods

Commercial starting materials were used without further purification. 2,2′bipyridine (Aldrich), 4,4′-dimethyl-2,2′-bipyridine (Aldrich), 5-Chlorothiophen-2-carboxylic acid (Hoechst Aktiengesellschaft), methanol (Qualigens, India), 5-Bromothiophene-2-carboxylic acid(Aldrich), Pb(CH_3_COO)_2_.3H_2_O were used. IR spectra of the complex in region 400-4000 cm^-1^ were recorded as pressed disks (1% by weight in KBr) on a Shimadzu FT IR spectrophotometer (Additional file 1). ^1^H-NMR and ^13^C-NMR spectra were recorded with a Bruker spectrometer at 400 MHz in [D6] DMSO (Additional file 1). CHNS analysis was carried out using Elementar Vario EL III in solid state (Table 5). The fluorescent properties were studied in solid state on a HITACHI spectrofluorimeter in solid state at room temperature. Both the excitation slit and emission slit were 5 nm. Thermal stability studies were carried out on a STA 409 PL Luxx thermal analyzer at a heating rate of 10°C/min under nitrogen atmosphere.

**Table 5:** Color, elemental analyses and stoichiometries of the Lead(II) complexes

**Complex**	**Color**	**Found (calculated) (%)**			
		**C**	**H**	**N**	**S**
[Pb(BPY)(5-CTPC)2]	Pale yellow	35.12	2.25	4.19	9.03
		(34.89)	(2.05)	(4.07)	(9.31)
[Pb(4-BPY)(5-CTPC)2]	Pale yellow	36.50	2.85	3.95	8.89
		(36.87)	(2.53)	(3.91)	(8.95)
[Pb2(PHEN)2(5-CTPC)4]	Yellow	37.58	1.96	3.85	9.03
		(37.08)	(1.98)	(3.93)	(9.00)
[Pb(4-BPY)(5-BTPC)2]	Yellow	32.95	2.25	3.55	8.00
		(32.84)	(2.13)	(3.48)	(7.97)
[Pb2(PHEN)2(5-BTPC)2(ACE)2]	Pale yellow	34.82	2.38	4.65	4.80
		(34.87)	(2.31)	(4.28)	(4.90)

### Synthesis of [Pb(BPY)(5-CTPC)_2_] (1)

**Scheme 2:**
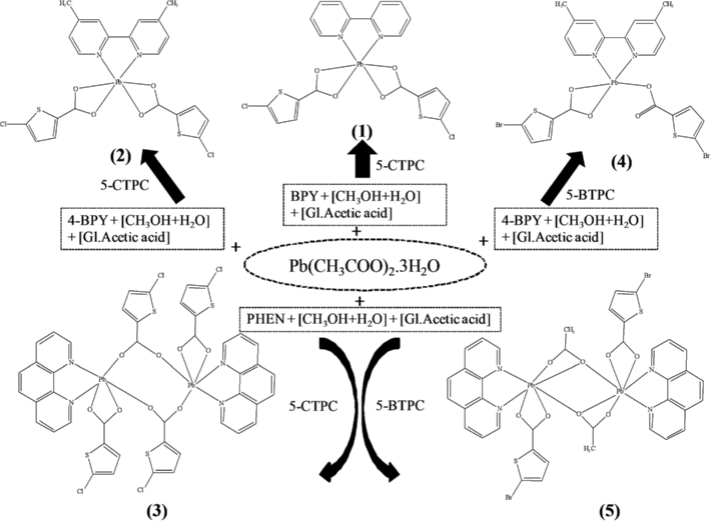
Preparation of complexes 1 – 5.

A solution of Pb(CH_3_COO)_2_.3H_2_O (0.098 g) in 10 ml of (1:1) CH_3_OH/H_2_O mixture was stirred over a hot plate magnetic stirrer for half an hour and 5-CTPC (0.0833 g) dissolved in 10 ml of CH_3_OH was added to it. The mixture was stirred for an additional of 2 hours. A yellow coloured solution was formed. About (0.0442 g) of (2-2′-bipyridine) was dissolved in 10 ml of hot water and added to the reaction mixture; to this solution about (5 ml) of glacial acetic acid was added. The mixture was stirred for 3 hours (Scheme 2). The dirty white precipitate was filtered off and the resulting pale yellow solution was kept for slow evaporation. Crystals were deposited at room temperature from the saturated solution. After 3 days, pale yellow coloured crystals suitable for X-ray diffraction were obtained. The crystals were filtered and washed with small portions of methanol and were dried in air (yield 75% based on Pb). IR selected bands (cm^–1^):1685(s), 1560(s), 1431(s), 1107(s), 994(s), 842(s), 794(s), 762(s), 723(s), 513(m). ^11^H NMR (400 MHz, DMSO): δ 9.32 (d, *J* = 4.8 Hz, 1 H), 8.69 (d, *J* = 6.4 Hz, 2 H), 8.38 (d, *J* = 10.0 Hz, 1 H), 8.11 (s, 1H), 7.95-7.94 (m, 2H), 7.45 (t, 1H), 7.28 (d, *J* = 4 Hz, 2 H), 7.08 (d, *J* = 4.0 Hz, 2 H) ppm; ^13^C NMR (100 MHz, DMSO): δ 166.3, 155.2, 150.0, 149.2, 144.9, 144.1, 137.7, 137.3, 131.3, 129.5, 129.3, 127.7, 127.0, 124.22, 124.17, 120.4 ppm.

### Synthesis of [Pb(4-BPY)(5-CTPC)_2_] (2)

The structure of complex (1) inspired us to design the preparation of complex (2) with same chelating mode using the 4,4′-dimethyl-2,2′-bipyridine ligand. The procedure of preparation of (2) is similar to (1). Instead of 2-2′-bipyridine, 4,4′-dimethyl-2,2′-bipyridine was used (Scheme 2) (yield 62% based on Pb). IR selected bands (cm^–1^): 3435 (m), 1432 (s), 1387(s), 1310(m), 1108(m), 1056(m), 996(s), 921(s), 835(s), 798(s), 767(s), 513(m),. ^1^H NMR (400 MHz, DMSO): δ 8.53 (d, *J* = 4.8 Hz, 2 H), 8.22 (s, 2 H), 7.32-7.27 (m, 4 H), 7.11 (s, 2H), 2.40 (s, 6 H) ppm; ^13^C NMR (100 MHz, DMSO): δ 166.4, 155.0, 148.9, 147.9, 144.0, 131.5, 129.7, 127.7, 124.9, 121.3, 20.7 ppm.

### Synthesis of [Pb_2_(PHEN)_2_(5-CTPC)_4_] (3)

The structures of complexes (1) and (2) inspired us to design the preparation of compound (2) with same chelating mode using 1,10-phenanthroline ligand. The procedure of preparation of (3) is similar to that of (1). Instead of 2-2′-bipyridine, 1,10-phenanthroline was used (Scheme 2) (yield 70% based on Pb). IR selected bands (cm^–1^):3047(m), 1557(s), 1372(s), 1205(m), 1138(s), 1105(s), 1055(s), 994(s), 860(s), 843(s), 798(s), 767(s), 725(s), 666(s), 511(m). ^1^H NMR (400 MHz, DMSO): δ 9.20 (d, *J* = 5.6 Hz, 2 H), 8.59 (d, *J* = 9.6 Hz, 2 H), 8.04 (s, 2 H), 7.90-7.87 (m, 2 H), 7.33 (d, *J* = 3.6 Hz, 1 H), 7.06 (d, *J* = 4.0 Hz, 1 H) ppm; ^13^C NMR (100 MHz, DMSO): δ 163.8, 149.9, 145.2, 136.9, 132.7, 131.0, 128.8, 127.9, 126.8, 123.7 ppm.

### Synthesis of [Pb(4-BPY)(5-BTPC)_2_] (4)

The procedure of preparation of (4) is similar to (2). Instead of 5-CTPC, 5-BTPC was used (Scheme 2) (yield 53% based on Pb). IR selected bands (cm^–1^): 1591(s), 1418(s), 990(s), 825(s), 764(s), 681(s), 514(s), . ^1^H NMR (400 MHz, CDCl_3_): δ 8.56 (d, *J* = 4.8 Hz, 1 H), 8.21 (s, 1 H), 7.15 (d, *J* = 4.8 Hz, 1 H), 2.44 (s, 3 H), 1.58 (s, 3 H), 1.25 (s, 2 H) ppm; ^13^C NMR (100 MHz, CDCl_3_): δ 148.9, 148.2, 124.7, 122.1, 21.2 ppm.

### Synthesis of [Pb2(PHEN)_2_(5-BTPC)_2_(ACE)_2_] (5)

The procedure of preparation of (5) is similar to (3). Instead of 5-CTPC, 5-BTPC was used (Scheme 2) (yield 67% based on Pb). IR selected bands (cm^–1^): 3388(w), 1562(s), 1358(s), 1098(s), 967(s), 843(s), 727(s), 766(s), 669 512(m). ^1^H NMR (400 MHz, CDCl_3_): δ 9.24-9.23 (m, 2 H), 8.28 (d, *J* = 9.6 Hz, 3 H), 7.81-7.66 (m, 4 H), 1.73 (s, 3 H), 1.25 (s, 1 H) ppm; ^13^C NMR (100 MHz, CDCl_3_): δ 150.3, 146.2, 136.1, 128.7, 126.6, 123.2 ppm.

### Single crystal X-ray structure analysis

Intensity data sets were collected at room temperature, on a BRUKER SMART APEXII CCD [56] area-detector diffractometer equipped with graphite monochromated Mo Kα radiation (λ = 0.71073 Å). The data were reduced by using the program SAINT [56] and empirical absorption corrections were done by using the SADABS [56]. The structures were solved by direct methods using SHELXS-97 [57] and subsequent Fourier analyses, refined anisotropically by full-matrix least-squares method using SHELXL-97 [57] within the WINGX suite of software, based on F2 with all reflections. All carbon hydrogens were positioned geometrically and refined by a riding model with U_iso_ 1.2 times that of attached atoms. All non H atoms were refined anisotropically. The molecular structures were drawn using the ORTEP-III [58], POV-ray [59] and MERCURY [60]. Crystal data and the selected parameters for complexes 1-5 were summarized in (Tables 3 and 6) respectively. Selected hydrogen bonding geometries are listed in (Table 2). The crystals remained stable throughout the data collection.

**Table 6:** Selected bond lengths (Å) and bond angles (°) for complexes 1-5

**Selected bonds**	**Value (Å)**	**Selected angles**	**(°)**	**Selected bonds**	**Value (Å)**	**Selected angles**	**(°)**	**Selected bonds**	**Value (Å)**	**Selected angles**	**(°)**
Complex 1				Complex 2				Complex 4			
Pb01-O1	2.532(2)	O1-Pb01-O2	51.55(6)	Pb1-O1	2.406(2)	O1-Pb1-O2	46.69(7)	Pb1-O1	2.407(3)	O1-Pb1-O4	120.61(9)
Pb01-O2	2.586(2)	O1-Pb01-O3	85.29(6)	Pb1-O2	3.019(2)	O1-Pb1-O3	120.44(7)	Pb1-O3	2.349(2)	O1-Pb1-N1	135.72(9)
Pb01-O3	2.518(2)	O1-Pb01-O4	134.86(6)	Pb1-O3	2.719(2)	O1-Pb1-O4	82.29(7)	Pb1-O4	2.718(4)	O1-Pb1-N2	75.88(7)
Pb01-O4	2.624(2)	O1-Pb01-N1	77.61(7)	Pb1-O4	2.3469(19)	O1-Pb1-N1	134.83(7)	Pb1-N1	2.631(3)	O3-Pb1-O4	51.45(9)
Pb01-N1	2.481(2)	O1-Pb01-N2	121.56(8)	Pb1-N1	2.626(2)	O1-Pb1-N2	75.57(7)	Pb1-N2	2.585(3)	O3-Pb1-N1	80.89(10)
Pb01-N2	2.595(2)	O2-Pb01-O3	132.41(7)	Pb1-N2	2.593(2)	O2-Pb1-O3	88.10(6)			O3-Pb1-N2	86.18(8)
		O2-Pb01-O4	152.80(7)			O2-Pb1-O4	83.59(6)			O4-Pb1-N1	77.42(10)
		O2-Pb01-N1	75.07(7)			O2-Pb1-N1	162.85(6)			O4-Pb1-N2	126.01(8)
		O2-Pb01-N2	75.97(7)			O2-Pb1-N2	122.19(6)			O1-C1-O2	125.1(3)
		O3-Pb01-O4	51.14(6)			O3-Pb1-O4	51.33(7)				
		O3-Pb01-N1	77.00(7)			O3-Pb1-N1	77.62(7)				
		O3-Pb01-N2	123.39(8)			O3-Pb1-N2	126.66(6)				
		O4-Pb01-N1	81.02(7)			O4-Pb1-N1	80.09(7)				
		O4-Pb01-N2	82.14(7)			O4-Pb1-N2	86.66(7)				
		N1-Pb01-N2	64.29(8)			N1-Pb1-N2	62.21(7)				
Complex 3				Complex5							
Pb1-O1	2.453(3)	O1-Pb1-O3	82.02(10)	Pb1-O1	2.749(5)	O1-Pb1-O2	50.02(12)				
Pb1-O2i	2.741(3)	O1-Pb1-O4	78.81(10)	Pb1-O2	2.335(3)	O1-Pb1-O3	119.74(11)				
Pb1-O3	2.333(2)	O1-Pb1-N1	135.32(11)	Pb1-O3	2.633(5)	O1-Pb1-O4	82.56(12)				
Pb1-O4	2.816(3)	O1-Pb1-N2	77.26(11)	Pb1-O4	2.824(3)	O1-Pb1-N1	79.17(12)				
Pb1-N1	2.604(3)	O1-Pb1-O2^i^	146.86(10)	Pb1-N1	2.608(4)	O1-Pb1-N2	120.32(11)				
Pb1-N2	2.612(3)	O3-Pb1-O4	49.97(9)	Pb1-N2	2.577(4)	O1-Pb1-O1^i^	63.24(11)				
Pb1 -Pb1^i^	3.7020(2)	O3-Pb1-N1	75.55(9)	Pb1 -O1 ^i^	2.909(5)	O2-Pb1-O3	87.25(12)				
		O3-Pb1-N2	86.32(10)			O2-Pb1 -O4	83.42(10)				
		O2i-Pb1-O3	102.13(10)			O2-Pb1 -N1	79.52(10)				
		O4-Pb1-N1	112.38(10)			O2-Pb1 -N2	77.27(12)				
		O4-Pb1-N2	132.48(10)			O1a-Pb1-O2	112.45(13)				
		O2i-Pb1-O4	79.23(10)			O3-Pb1 -O4	47.69(13)				
		N1-Pb1-N2	63.34(10)			O3-Pb1 -N1	139.85(12)				
		O2i -Pb1-N1	76.46(10)			O3-Pb1 -N2	76.38(11)				
		O2i-Pb1-N2	135.46(10)			O1-C1-O2	121.9(5)				
						O1a-Pb1-O3	140.20(9)				
						O4-Pb1 -N1	160.44(13)				
						O4-Pb1 -N2	121.44(12)				
						O3-C3-O4	125.2(5)				
						O1a-Pb1-O4	98.61(11)				
						N1-Pb1 -N2	63.81(14)				

Complex 3 symmetry code (i):-x,1-y,1-z Complex 5 symmetry code (i): 2-x,1-y,1-z.

## Conclusions

A series of novel Pb(II) complexes in concert with 5-CTPC, 5-BTPC and corresponding bidentate chelating N.N’ ligands have been synthesized and characterized. There is significant structural diversity even though, in all the complexes the molar ratio of Pb, carboxylate, N,N-chelating ligand (ie, 1:2:1) is the same. The complexes 1, 2, 4 are mononuclear and are structurally diverse compared with 3, 5 which are dinuclear in nature. The observation of structures 2, 4 and 3, 5 show the structural changes made just chloro/bromo substituent of the thiophene ring. In addition to noncovalent interaction like C-H…O, which is the reason for assembly of primary motifs; various other interactions like X…π, H…X, (X = Cl/Br) add additional support in organizing these supermolecules in to extended architectures. It is noteworthy that the organic ligands not only serve as a space filling component but they are also involved in structural propagation either by π- π stacking or C-H… π stacking interactions. Complexes 1-5 exhibit strong emissions and may be potential materials for emitting diode devices. However this work not only shows the influence of replacement of different ligands on the structure but also it illustrates the effects of substituent’s on the ligands on the crystal structure and geometry of the central metal ion, which also provides valuable instruction in design of coordination complexes with desired supramolecular architectures.

## Competing interests

Both authors declare that they have no competing interests.

## Authors’ contributions

This work was prepared in the research group of PTM. He proposed the work and drafted the manuscript. SJJ participated in the design and presiding the experiments, collected the X-ray data and drafted the manuscript. Both authors read and approved the final manuscript.

## Supplementary Material

Additional file 1 contains the IR, NMR spectra and the TGA curves for complexes (1-5). Click here for file
