# Supplementary material for: Supramolecular architectures and structural diversity in a series of lead (II) Chelates involving 5-Chloro/Bromo thiophene-2-carboxylate and N,N’-donor ligands
Source: Chem Cent J. 2013 Aug 15;7:139. doi: 10.1186/1752-153X-7-139 (PMC3766206; doi:10.1186/1752-153X-7-139)
Supplement: Additional file 1 — contains the IR, NMR spectra and the TGA curves for complexes (1-5). [file 1752-153X-7-139-S1.doc]

**IR- Complex 1**

**IR- Complex 2**

**IR- Complex 3**

**IR- Complex 4**

**IR- Complex 5**

**Complex 5 ^1^H NMR**


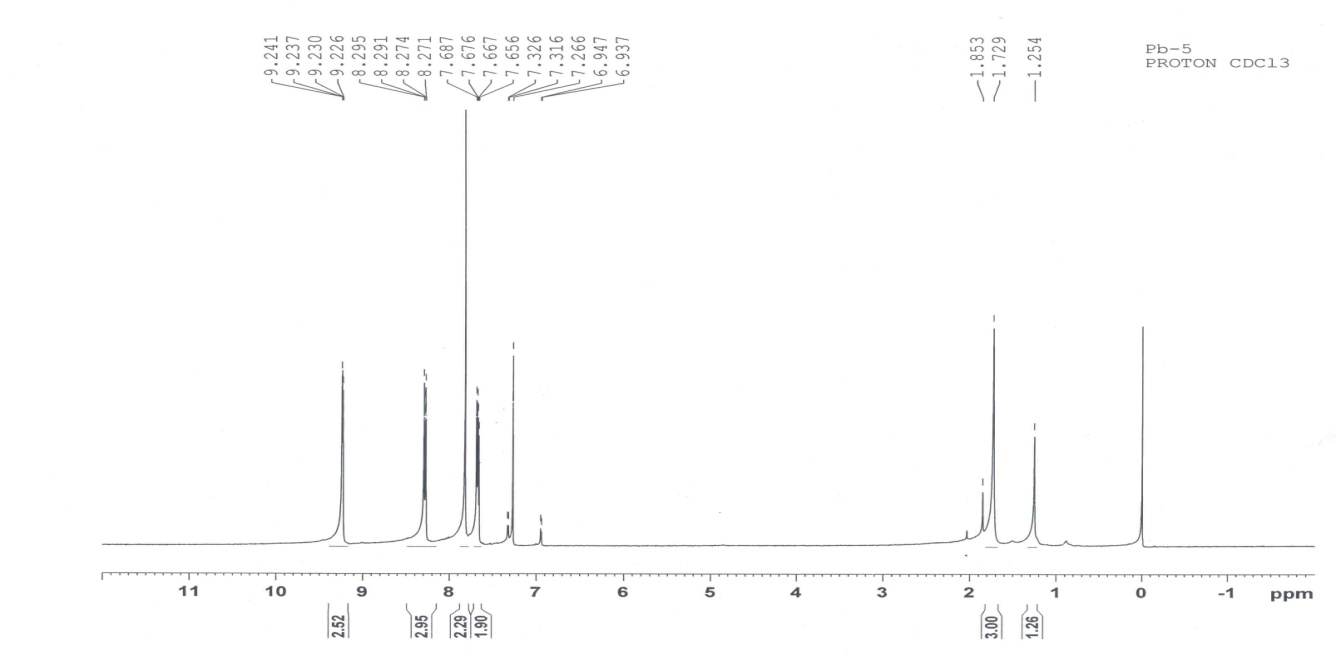
 **Complex 5 ^13^C NMR**


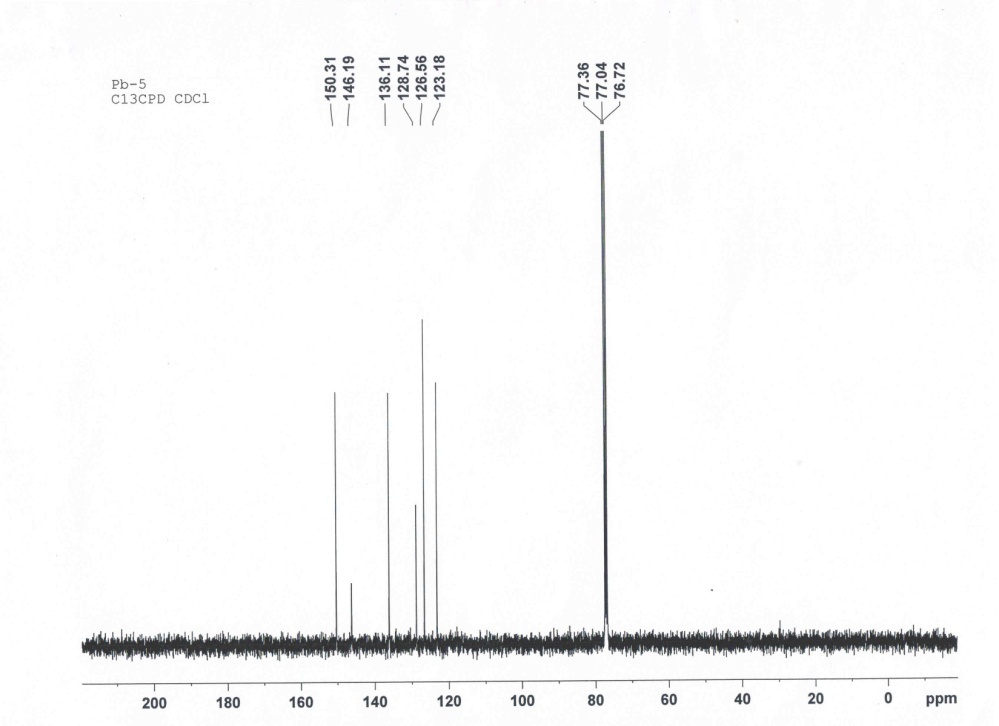


**Complex 4 ^1^H NMR**


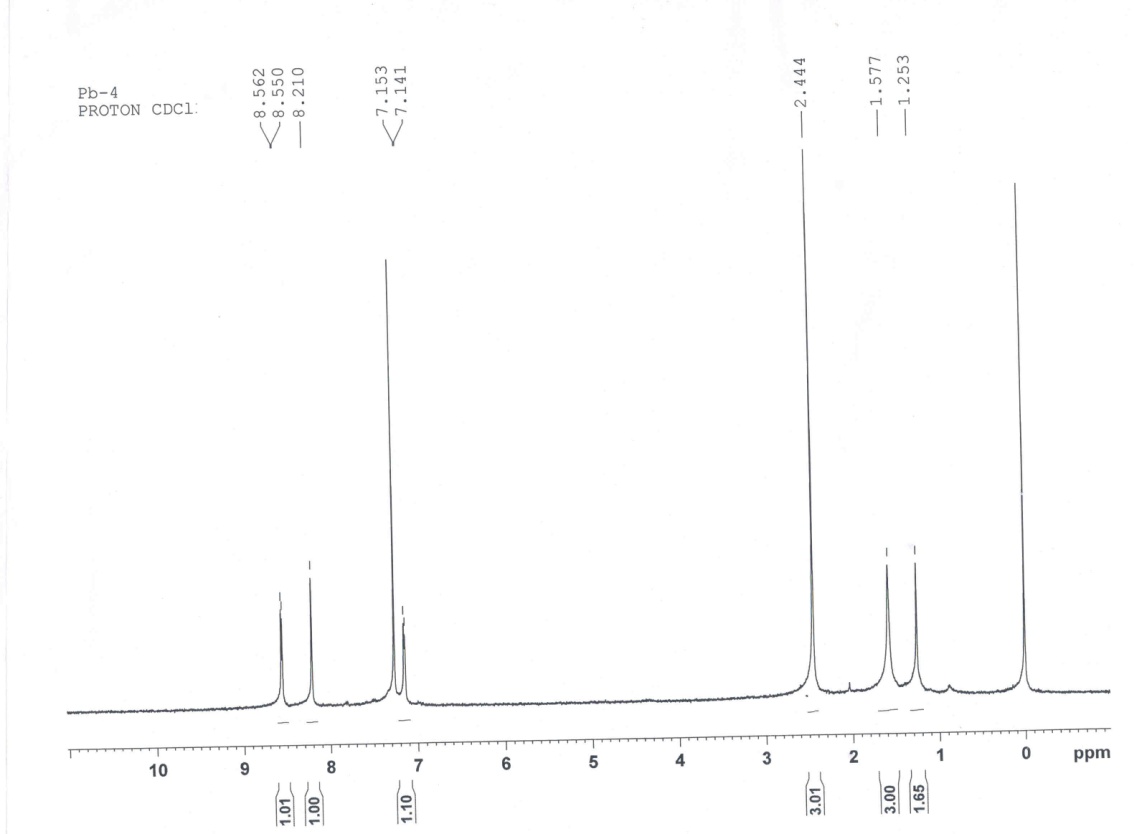


**Complex 4 ^13^C NMR**


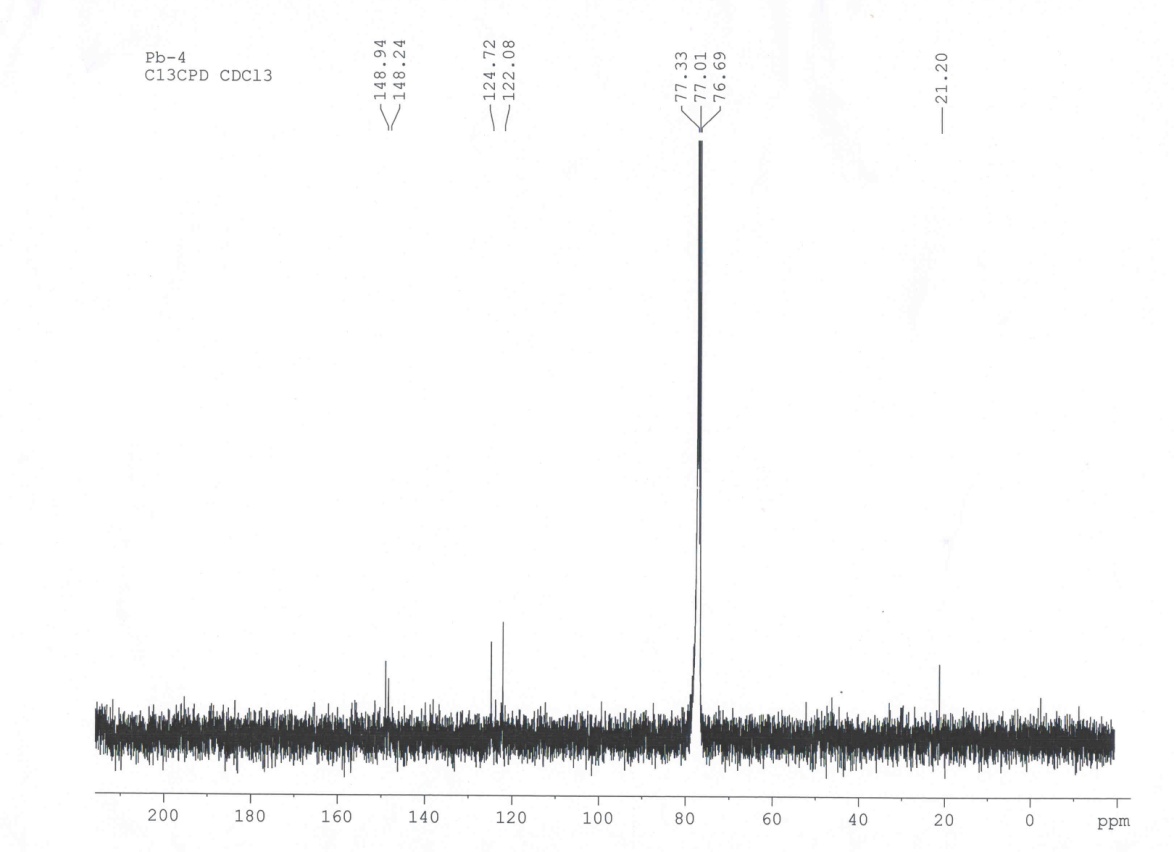


**Complex 3 ^1^H NMR**


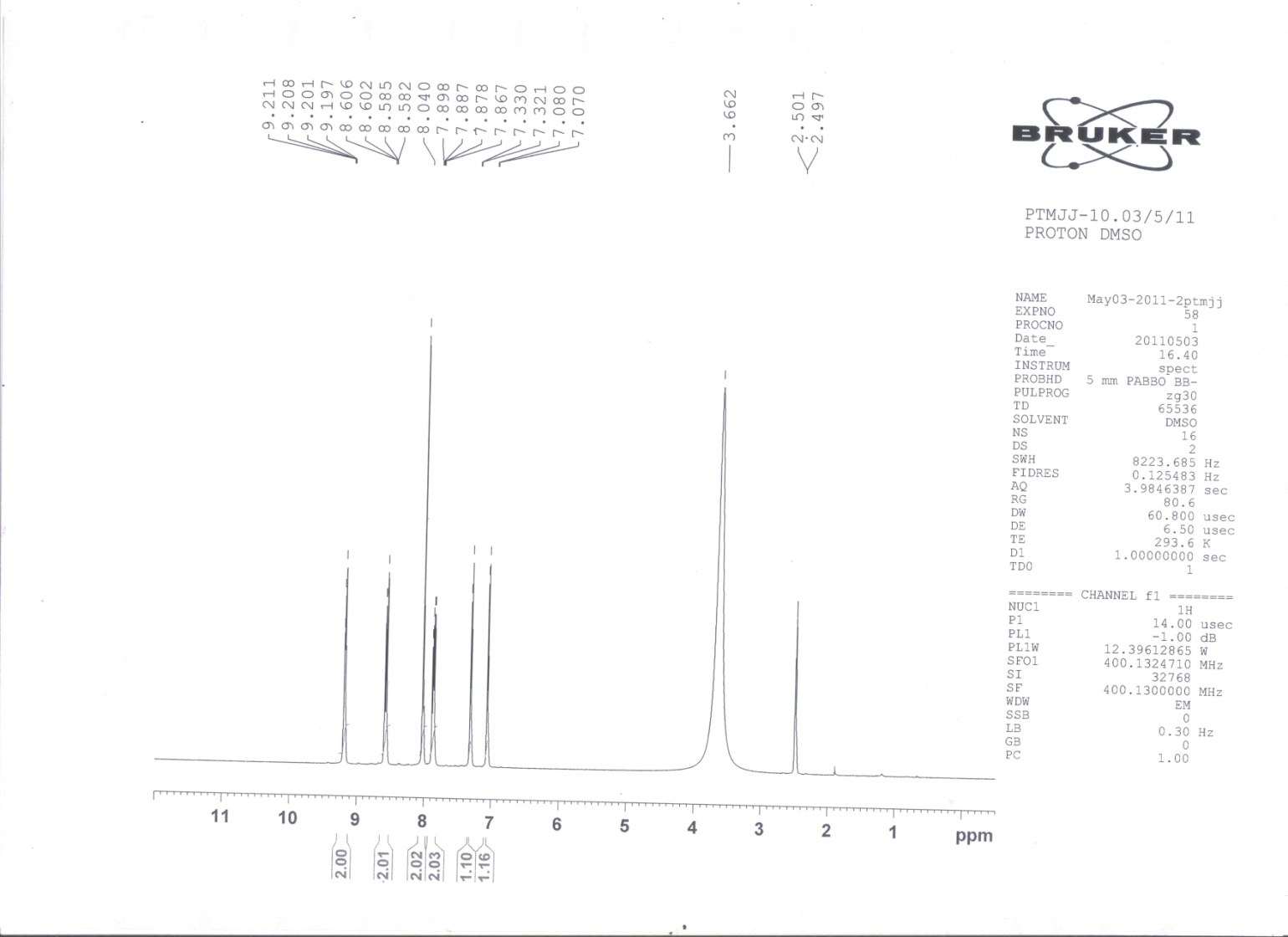


**Complex 3 ^13^C NMR**


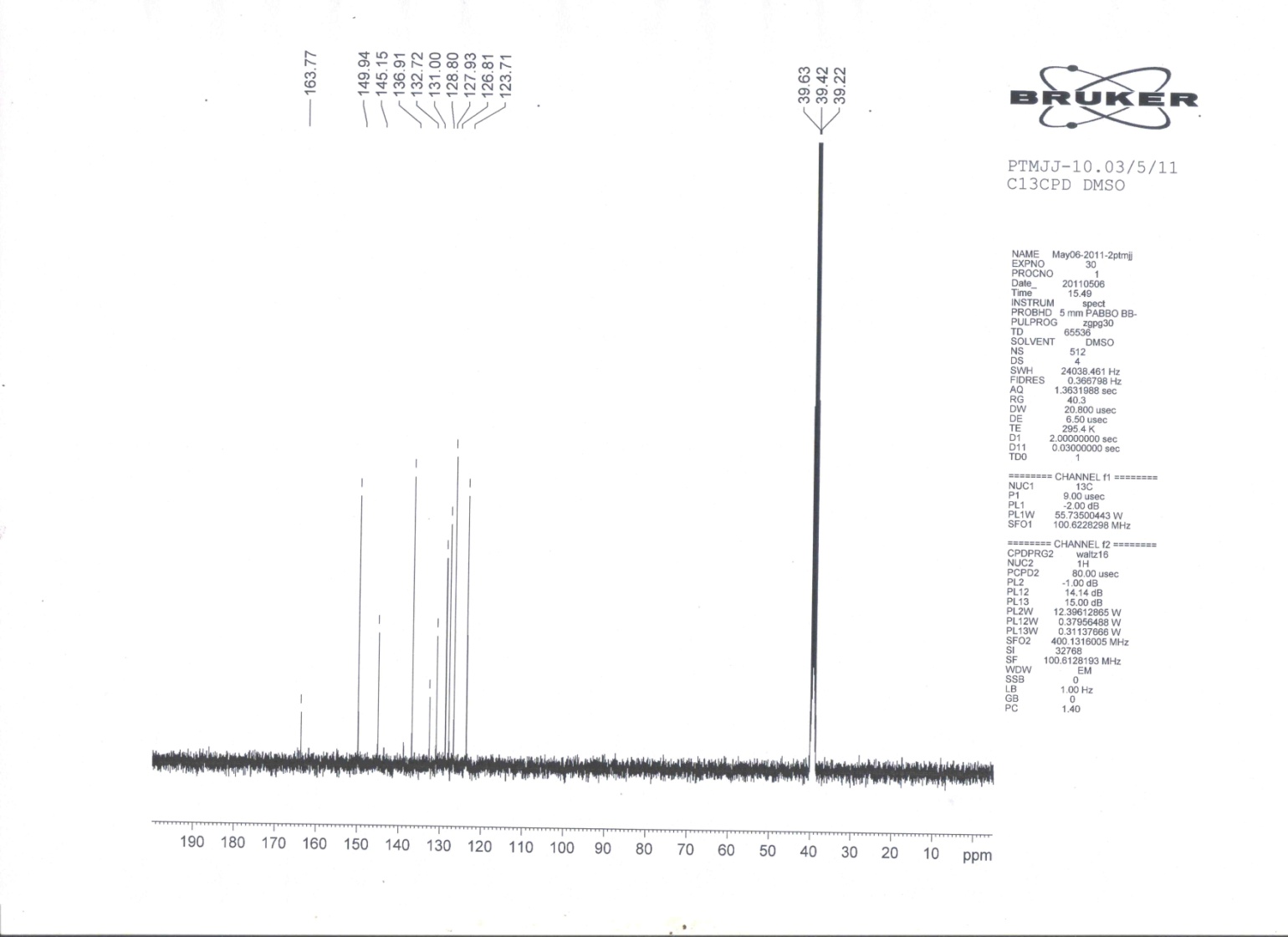


**Complex 2 ^1^H NMR**


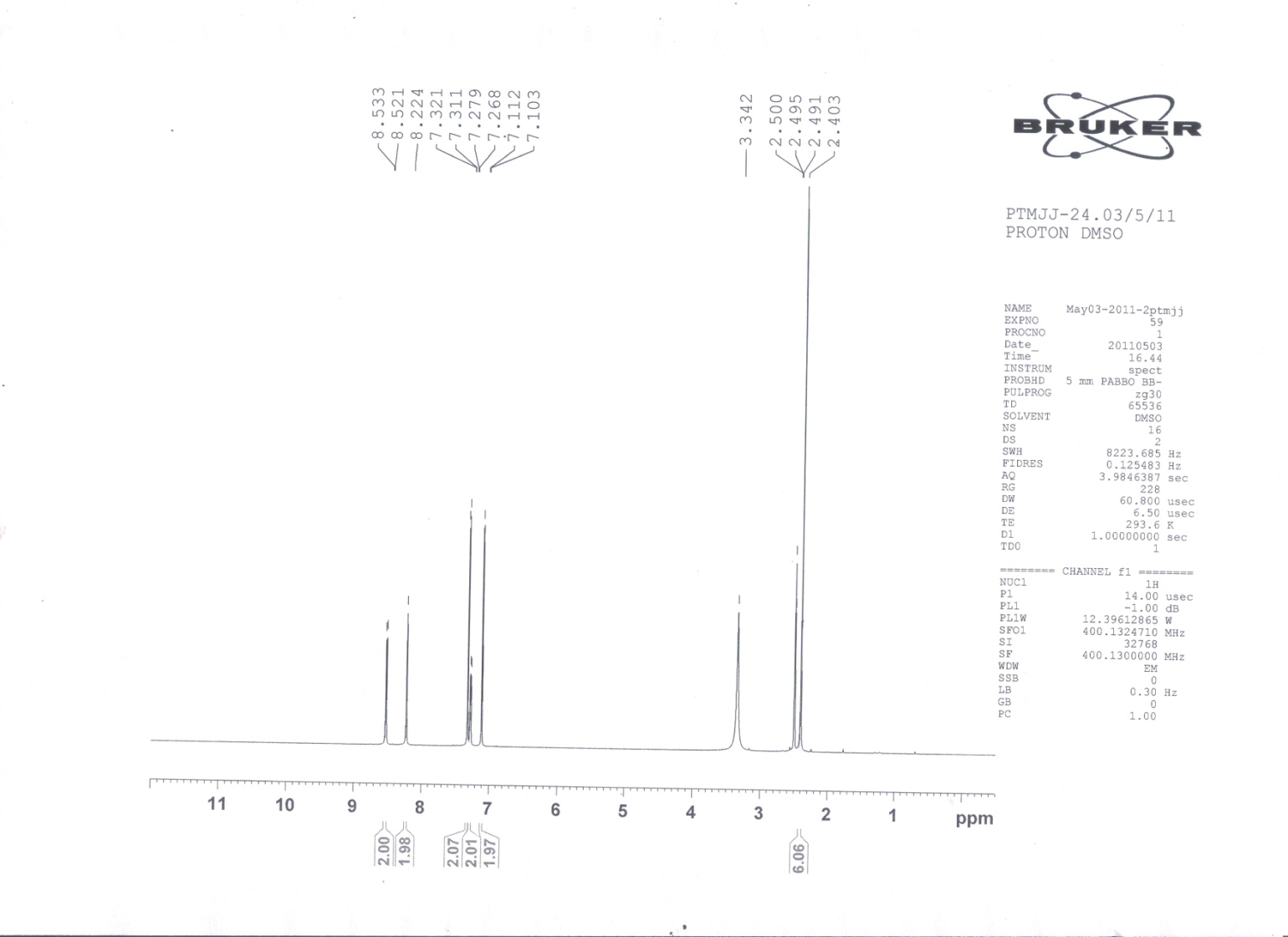


**Complex 2 ^13^C NMR**


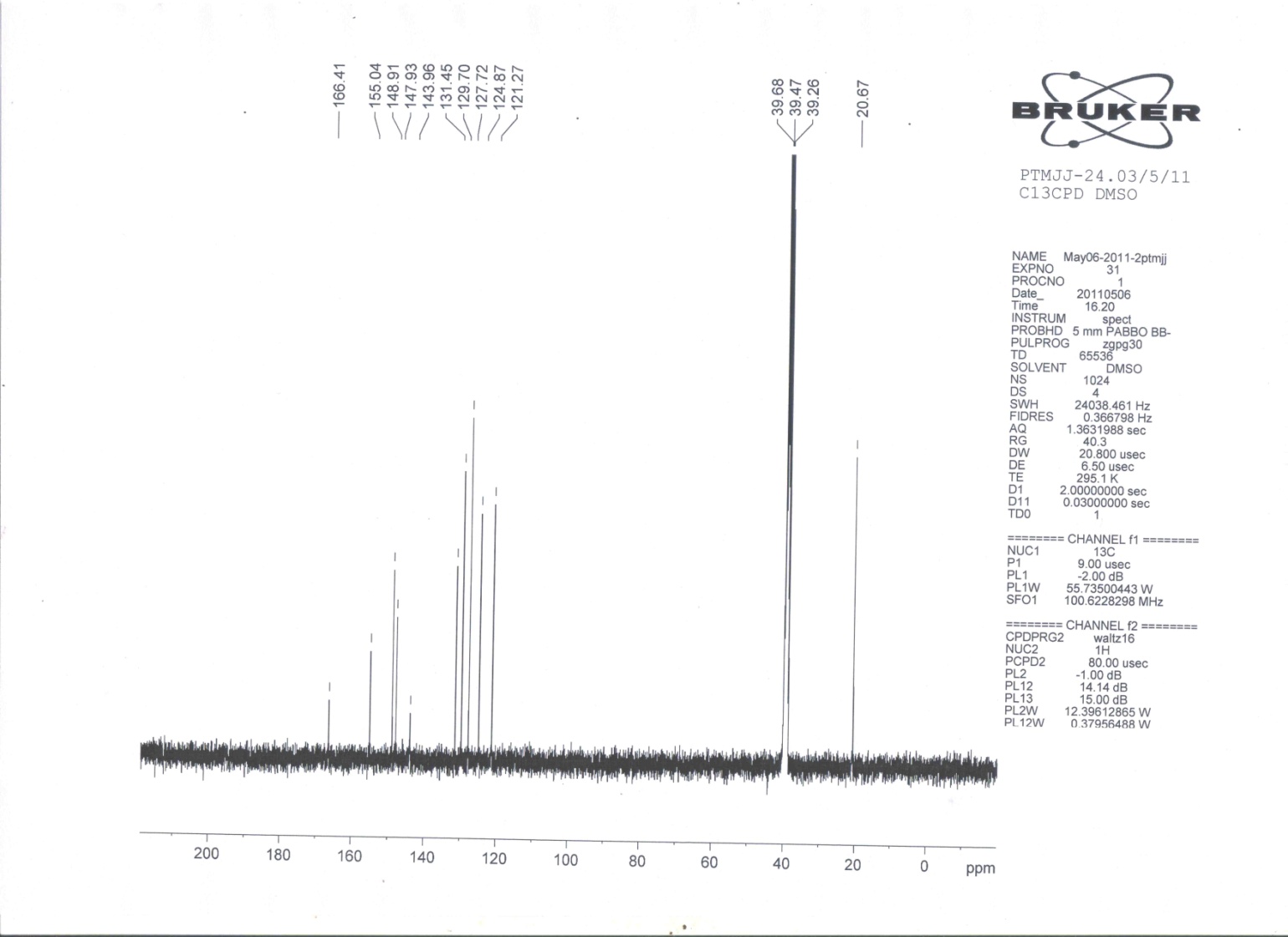


**Complex 1 ^1^H NMR**


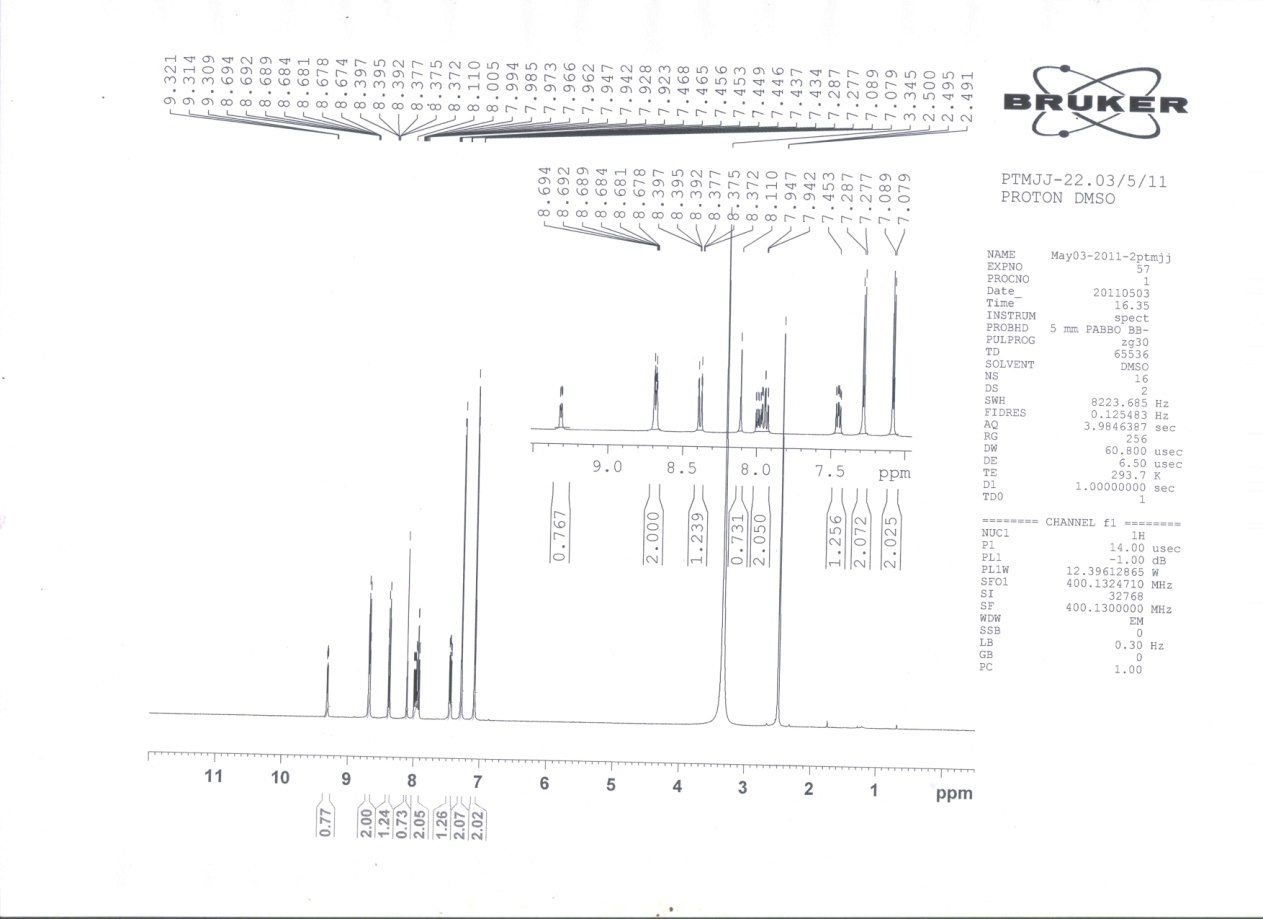


**Complex 1 ^13^C NMR**

**
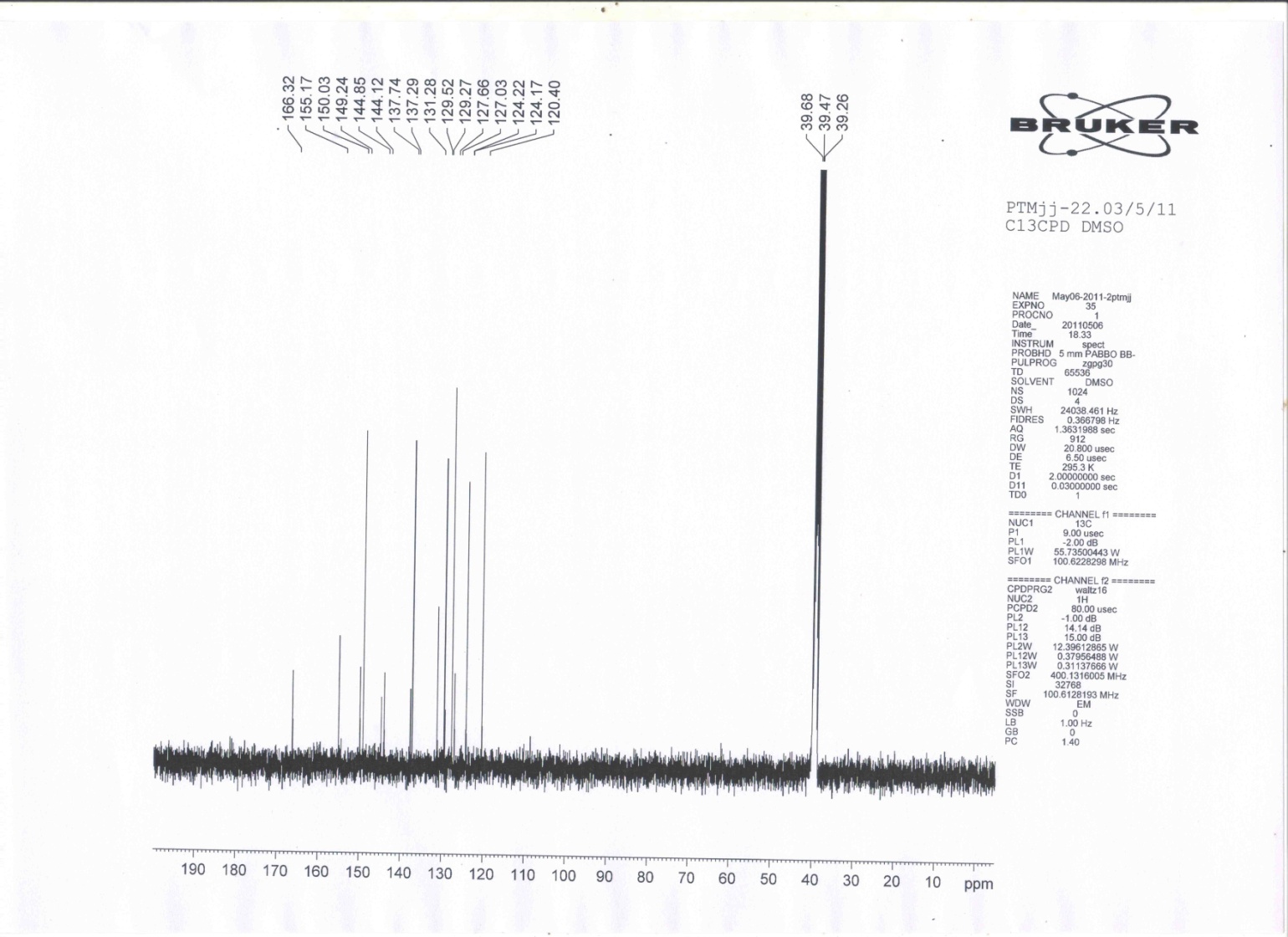
**

**TGA-DTA Complex 5**


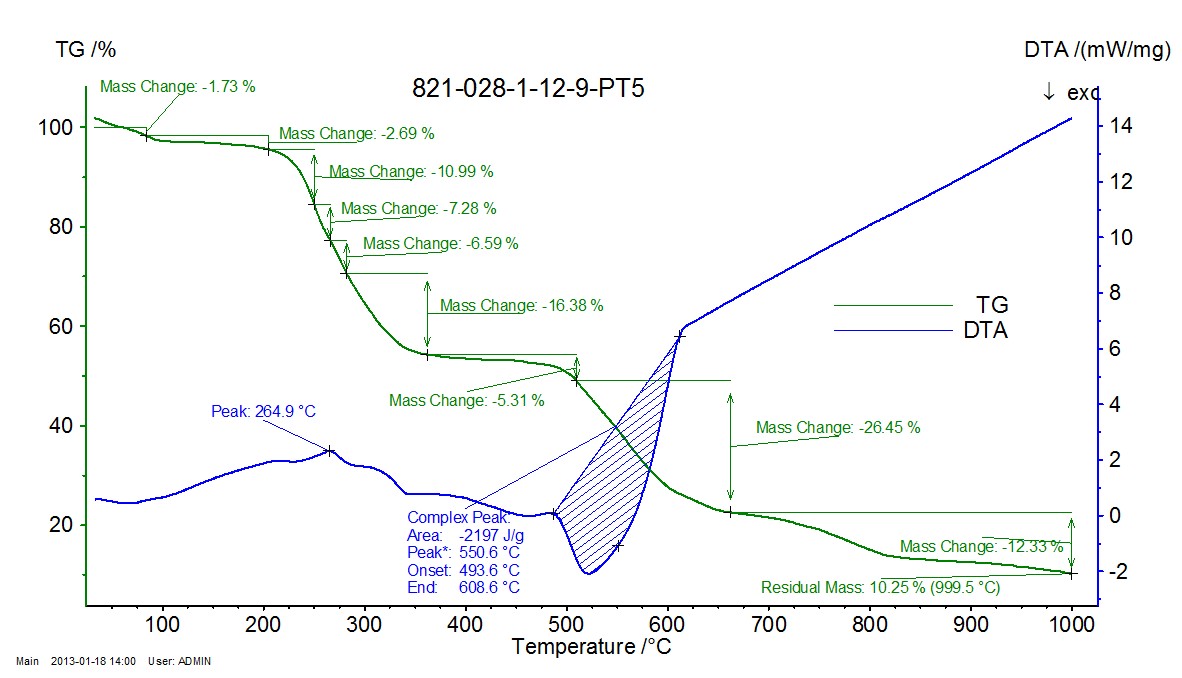


**TGA-DTA Complex 4**


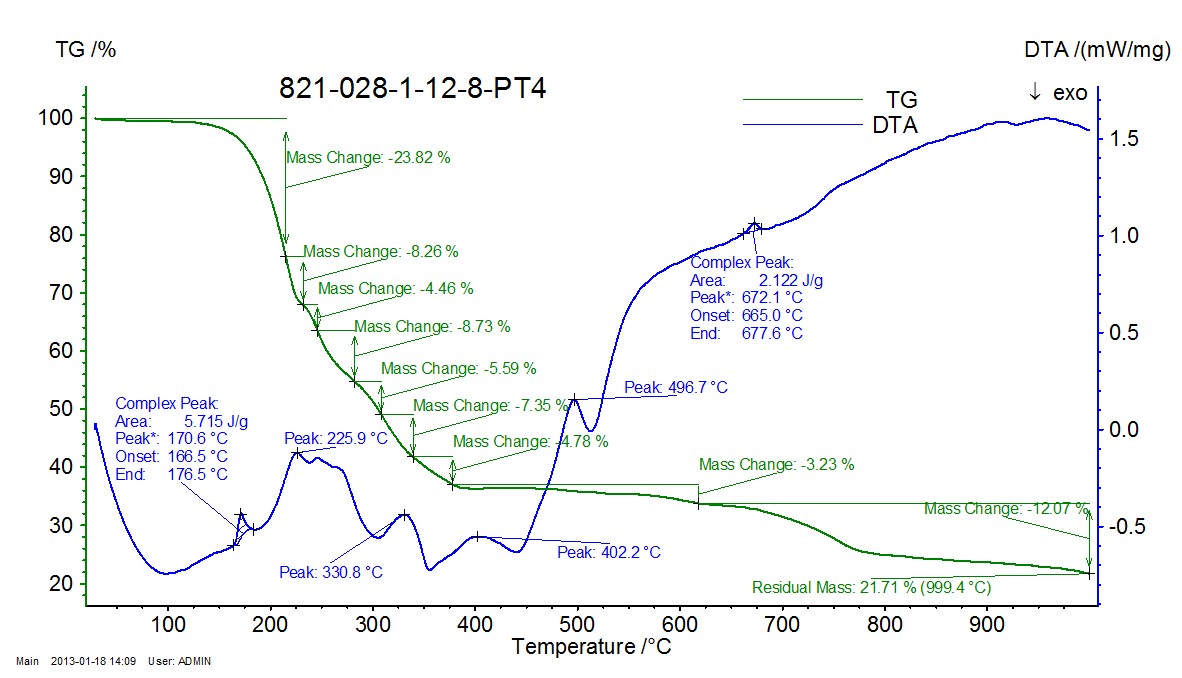


**TGA-DTA Complex 3**
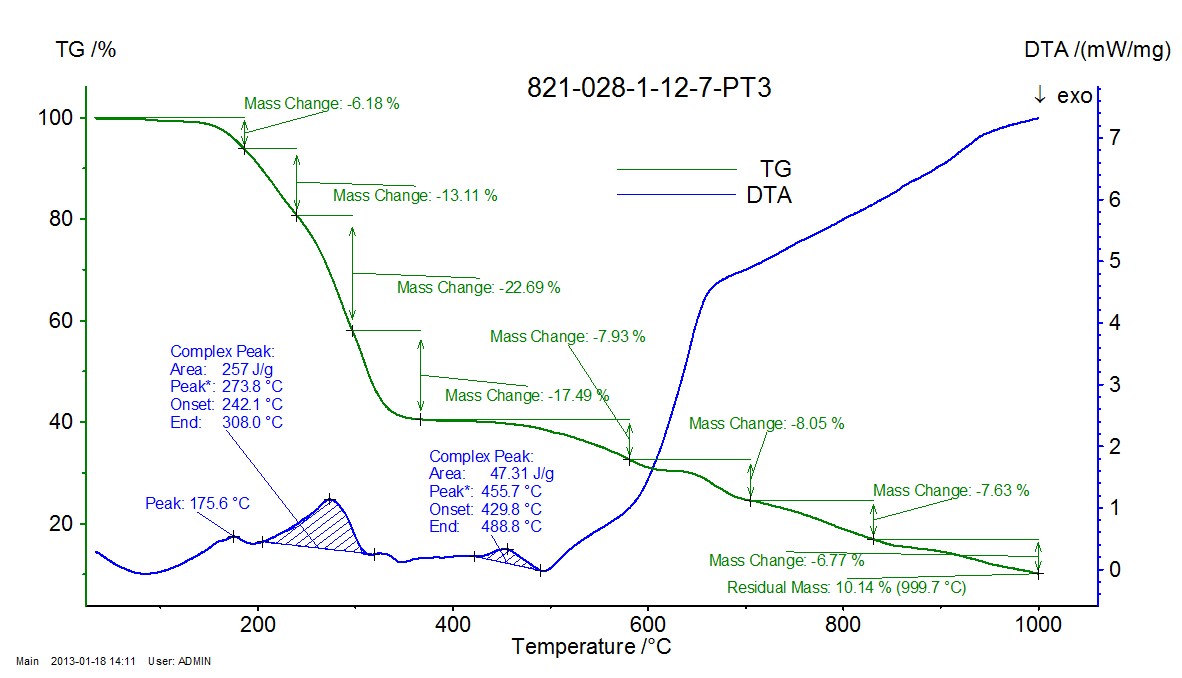


**TGA-DTA Complex 2**


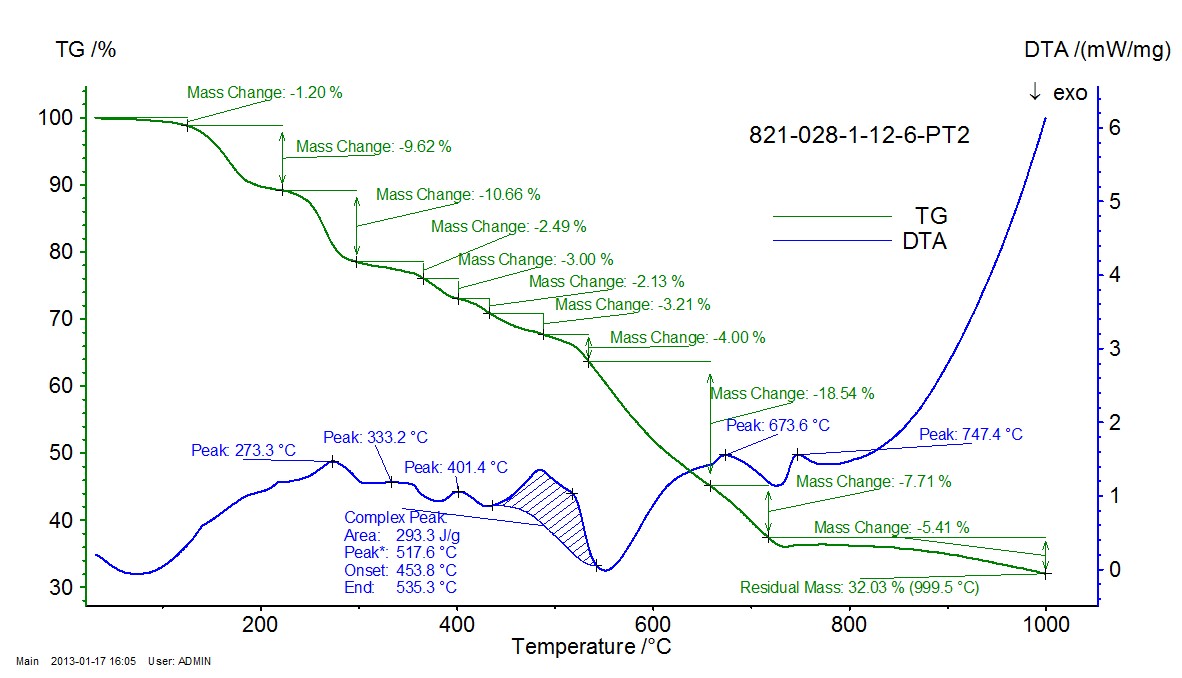


**TGA-DTA Complex 1**

**
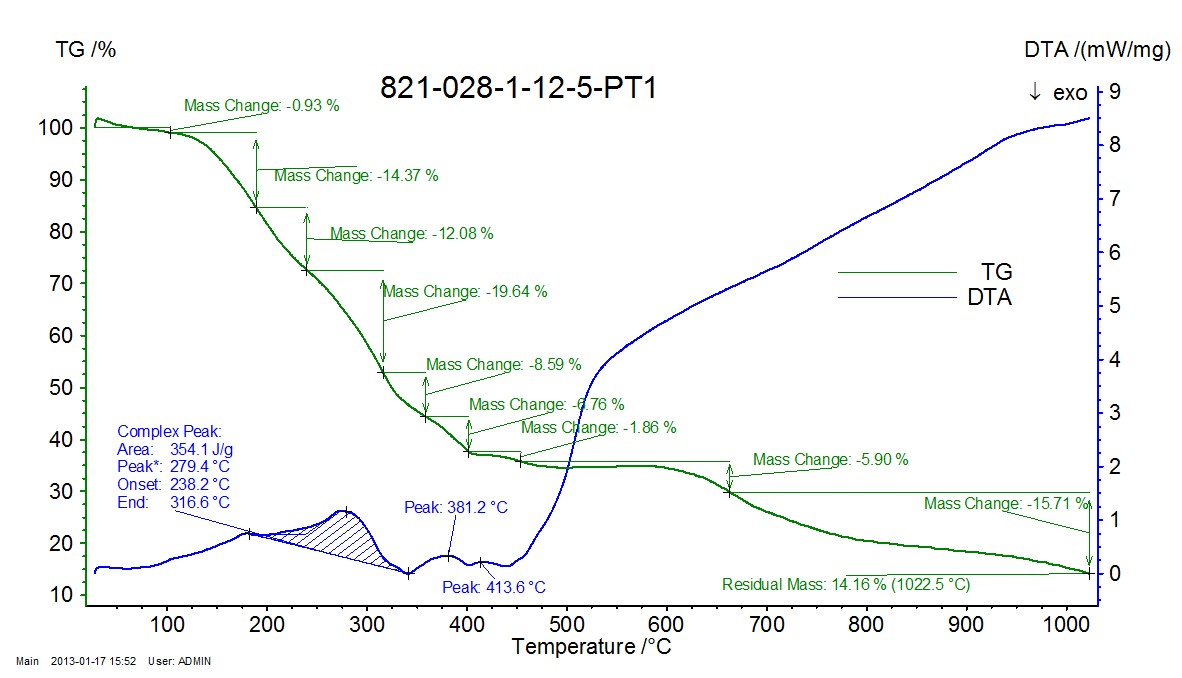
**
